# Risk Factors for Developmental Dysplasia of the Hip Before 3 Months of Age: A Meta-Analysis

**DOI:** 10.1001/jamanetworkopen.2024.56153

**Published:** 2025-01-24

**Authors:** Maria Tirta, Ole Rahbek, Søren Kold, Hans-Christen Husum

**Affiliations:** 1 Interdisciplinary Orthopaedics, Department of Orthopaedic Surgery, Aalborg University Hospital, Aalborg, Denmark

## Abstract

**Importance:**

Two meta-analyses published in 2012 found breech presentation, family history of developmental dysplasia of the hip (DDH), female sex, and primiparity to increase the risk of DDH. However, the DDH definition, reference tests, and the age of the examined children varied considerably, complicating the translation of those findings to current screening guidelines.

**Objective:**

To evaluate the association of previously proposed risk factors with the risk of sonography-verified DDH.

**Data Sources:**

A literature search strategy used MeSH (Medical Subject Heading) terms and text words associated with DDH and prognostic studies or risk factors. PubMed, Embase, and the Cochrane Library were searched on November 23, 2023, to identify the cohort.

**Study Selection:**

Criteria included articles that were in English and published from January 1, 1980, to November 23, 2023. Eligible studies included randomized clinical trials and cohort, case-control, and cross-sectional studies that involved participants younger than 3 months in whom a diagnosis of DDH was made by hip ultrasonography using the criterion standard Graf method, reported information on 1 or more of the proposed risk factors, and reported the final diagnosis.

**Data Extraction and Synthesis:**

Collected variables included an evaluated risk factor, the number of infants with a risk factor, the number of infants without risk factors, and the number of infants with and without DDH in the at-risk group and in the no-risk group. This study followed the Preferred Reporting Items for Systematic Reviews and Meta-Analyses (PRISMA) and Meta-Analysis of Observational Studies in Epidemiology (MOOSE) reporting guidelines.

**Main Outcomes and Measures:**

The main outcome measures were risk of DDH (using odds ratios [ORs]) for each of the risk factors.

**Results:**

Of 5363 studies screened, 20 studies comprising 64 543 infants were included. Breech presentation (OR, 4.15 [95% CI, 2.62-6.57]), family history of DDH (OR, 3.83 [95% CI, 2.05-7.15]), female sex (OR, 2.50 [95% CI, 1.74-3.59]), oligohydramnios (OR, 3.76 [95% CI, 1.66-8.53]), and high birth weight (OR, 2.00 [95% CI, 1.60-2.49]) were associated with a significant increased risk of DDH. Cesarean delivery, primiparity, multiple births, low birth weight, and prematurity were not associated with DDH risk. Heterogeneity was high (*I*^2^ > 70.00%) in all of the tested factors except high birth weight (*I*^2^ = 0%). The subgroup analysis was performed to investigate these heterogeneities.

**Conclusions and Relevance:**

The findings of this meta-analysis suggest that family history and breech presentation were associated with a significant increase in the risk of sonography-verified DDH in infants younger than 3 months, and a DDH risk increase of female sex was found to be lower than previously reported. A risk increase was detected for oligohydramnios, which has not been included in many screening programs and might also be considered as a referral criterion for hip ultrasonography.

## Key Points

**Question:**

In infants younger than 3 months, what factors are associated with risk of developmental dysplasia of the hip (DDH), defined as a Graf ultrasound classification of type IIb or higher?

**Findings:**

In this meta-analysis of 20 studies that included 64 543 infants, breech presentation and family history of DDH were the most significant risk factors for DDH, followed by oligohydramnios, female sex, and high birth weight.

**Meaning:**

Current screening guidelines highlighting breech presentation and family history as the main risk factors for referral criteria for hip ultrasonography were supported by the present findings, while oligohydramnios might also be considered as a referral criterion, as it demonstrated similar risk of DDH.

## Teaser

This meta-analysis evaluates whether the presence of a proposed risk factor is associated with increased risk of developmental dysplasia of the hip, as defined by Graf ultrasonography, in infants younger than 3 months.

## Introduction

Screening for developmental dysplasia of the hip (DDH) is widely implemented and can be generally divided into a universal ultrasonographic screening, in which all children are referred for hip ultrasonography (US), and selective screening, in which only children at risk of DDH are referred for hip US.^1^ The evaluation of DDH risk in selective screening is generally performed using clinical hip examinations and identification of risk factors for DDH. Many risk factors have been proposed with breech presentation and family history of DDH being the most used referral criteria.^2,3^ While female sex is recognized as a significant risk factor for DDH, it is not widely implemented as a referral criterion, possibly due to the logistical impact of screening all newborn females.^4^ Other suggested risk factors are oligohydramnios, multiple births, primiparity, clubfoot, prematurity, high birth weight, and cesarean delivery.^5^

In 2 meta-analyses published on this subject including all study types, breech presentation, female sex, and primiparity were found to increase the risk of DDH, while foot deformities, oligohydramnios, and multiple births were not found to increase the risk.^6,7^ However, the definition of DDH, diagnostic tests, and the age of the examined children varied considerably between these meta-analyses within their included studies, complicating the generalizability of resulting findings. A recent meta-analysis by Chen et al,^8^ which focused exclusively on cohort studies, included many different risk factors, but it also included diagnoses made through physical examinations, US, and x-rays, which introduced potential bias. This bias was further compounded by 1 study within the meta-analysis that included 807 889 male infants of the total 979 757 infants, which possibly skewed the overall findings. In addition, a great number of studies examining the correlation between possible risk factors and DDH have been published after 2012, highlighting the need for an updated and well-defined meta-analysis on the topic. The Graf classification is a US-based system used to assess hip development in infants, categorizing hip joints from type I (normal) to type IV (severely dysplastic or dislocated). Type IIb refers to hips with delayed acetabular development in infants 3 months or older.^9^

To limit the aforementioned heterogeneity, the aim of the present meta-analysis was to evaluate the association of previously proposed risk factors with the risk of sonography-verified DDH in infants younger 3 months. We sought to answer the following question for the aforementioned risk factors: in infants screened younger than 3 months, does the presence of a proposed risk factor increase the risk of DDH defined as a Graf US classification of type IIb or higher?

## Methods

This meta-analysis is reported according to the Preferred Reporting Items for Systematic Reviews and Meta-Analyses (PRISMA) and Meta-analysis of Observational Studies in Epidemiology (MOOSE) reporting guidelines. The study was registered with PROSPERO (registration number CRD42024505430).

### Selection Criteria

Eligible studies included infants younger than 3 months in whom a diagnosis of DDH was made by hip US using the Graf method and presented information on the presence of 1 or more of the proposed risk factors (breech presentation, female sex, family history of DDH, oligohydramnios, multiple births, birth weight, prematurity [<37 weeks’ gestation], primiparity, cesarian delivery, or clubfoot). To ensure comparability in DDH diagnoses across studies, we defined DDH as a Graf US classification of type IIb or higher.^9^ We therefore only included studies in which the Graf US classifications and/or alpha angles were reported. We allowed for some overlap of the 3-month age cutoff for studied infants if most infants in the included studies were younger than 3 months and did not significantly exceed this age limit. Cohort studies, randomized clinical trials, case-control studies, and cross-sectional studies within a time frame of January 1, 1980, to November 23, 2023, were included when full texts were available in English. We excluded studies that did not contain the raw information needed for the construction of 2-by-2 tables for risk factor analyses.

### Search Strategy

A literature search strategy was developed by H.-C.H. and a research librarian using MeSH (medical subject heading) terms and text words associated with DDH and prognostic studies or risk factors. Literature searches were performed through PubMed, Embase, and the Cochrane Library on November 23, 2023. Full search strings, accessed dates, and search results for 3 databases can be seen in eTable 1 in Supplement 1. The reference section of eligible studies was examined for relevant cross-references not apparent from the primary literature search, but no additional study was identified.

M.T. and H.-C. H. independently screened all titles and abstracts for eligible studies using the Rayyan online management program.^10^ In case of unresolvable disagreement, the full text was acquired and discussed once the primary screening was completed to reach consensus of eligibility. If agreement could not be reached, O.R. acted as the adjudicator, resolving any disagreements. None of the authors were blinded to study titles, institutions, or authors of screened studies. Full-text screening was independently performed by 2 authors (M.T. and H.-C. H.) with O.R. as the adjudicator in case of unresolvable disagreements. Duplicate cohorts were thoroughly scrutinized across all studies before the final list for the meta-analysis was confirmed.

### Data Extraction

Two authors (M.T. and H.-C. H.) independently extracted data from each selected study using a standardized data extraction tool, developed in Excel, version 15.16 (Microsoft Corp) by the authors to systematically capture relevant data. Prior to formal data extraction, the tool was piloted on a subset of studies to ensure consistency. M.T. and H.-C. H. then met to compare results, resolve any discrepancies, and refine the tool as necessary to standardize the extraction process across all studies. We collected data associated with risk of DDH diagnosis stratified by risk factor status, for all of the aforementioned examined risk factors. Collected variables included the evaluated risk factor, the number of infants with a risk factor, the number of infants without risk factors, and the number of infants with or without DDH in the at-risk group and in the no-risk group. For prematurity and birth weight, we additionally collected information on any study-specific definition of prematurity and birth weight. Additionally, we collected data on the study design, the year of publication, the number of patients and/or hips, the screening strategy (universal or selective), the time of screening, the prevalence of DDH in the study population (calculated by the authors if not reported directly), the definition of DDH, and the examined risk factors.

Since many of the included studies involved selective screening programs in which no infants were entirely without risk factors, we considered the absence of a specific risk factor as equivalent to having no risk factors. For example, an infant referred for a hip US due to breech presentation would be categorized in the no-risk group when evaluating the risk of family history, provided that a family history of DDH was not reported. Furthermore, as reporting on infants with multiple risk factors was not accounted for in most of the included studies, we assumed no effect modification or interaction of risk of DDH for infants with multiple risk factors. All data were extracted in a shared Excel, version 15.16 sheet constructed for the purpose of this study.

All studies that met the inclusion criteria underwent methodologic quality assessment using the QUADAS-2 tool for evaluating risk of bias in diagnostic accuracy studies.^11^ Each study was scored independently by M.T. and H.-C. H. on the following criteria: patient selection, conduct of the index test, conduct of the reference standard test, and flow and timing. Additionally, applicability for the research question was scored for patient selection, the index test, and the reference standard. Per the QUADAS-2 guidelines,^11^ study-specific signaling questions were developed for the present study; these were: Were children without risk factors included? (for the patient selection category), Were patients without any risk factors clearly labeled? (for the index test category), and Was a US image of the Graf examination produced to verify a correct procedure? (for the reference standard scoring category). The robvis tool was used to generate the table for risk of bias.^12^

### Statistical Analysis

The *Cochrane Handbook for Systematic Reviews of Diagnostic Test Accuracy* method was applied.^13^ Data were entered in contingency tables, and odds ratios (ORs) (95% CIs) were estimated for each study, as well as a pooled estimate, weighted by the sample size of each study. We calculated the summary effect sizes using a random-effects model. The random-effects model posits that the actual effect size fluctuates across studies, and the studies included represent a random sample of potential effect sizes. Consequently, the random-effects model accommodates variability, not only within studies but also between studies, resulting in a cautious estimate of summary statistics with broader than 95% CIs. Pooled proportions were computed, and forest plots were generated to visualize the data. Heterogeneity among studies was evaluated using Cochran *Q* and the *I*^2^ statistic. The threshold for statistical significance was set at *P* < .05. All statistical tests were 2-sided to account for both potential directions of the effect. The subgroup analysis and the sensitivity analysis were performed to investigate sources of heterogeneity. The parameters for the subgroup analysis were as follows: study design, time of screening, type of screening, definition of DDH, risk of bias, and counting of the participants (per hip per patient). eTable 3 in Supplement 1 presents how the variables were grouped to be used for the subgroup analysis.

A funnel plot was constructed to examine publication biases in which there were 10 or more studies, and an Egger test was used to assess funnel plot asymmetry and the small studies’ effect. Statistical significance was set at a 2-sided *P* < .05. Stata, version 18.0 (StataCorp LLC) was used for analyses and to derive forest and funnel plots.

The overall certainty of evidence was evaluated using the Grading of Recommendations, Assessment, Development, and Evaluation (GRADE) guidelines, which provide 4 possible ratings: high, moderate, low, and very low.^14^ The rating can be downgraded due to study limitations, inconsistency, indirectness, imprecision, and/or publication bias.

## Results

### Included Studies

**Figure 1.**
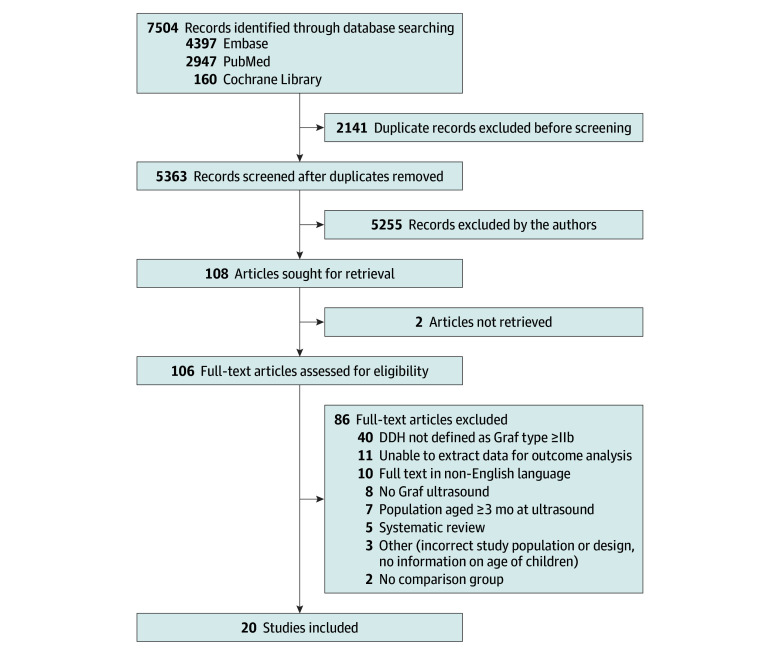
Flowchart of Included Studies DDH indicates developmental dysplasia of the hip.

The electronic search from the databases yielded 5363 studies after removing duplicate records, of which 106 were screened for full text. After the full-text manuscript review, 86 studies were excluded (eTable 2 in Supplement 1), and 20 studies were included (Figure 1), of which 14 were cohort studies,^15,16,17,18,19,20,21,22,23,24,25,26,27,28^ 5 were case-control studies,^29,30,31,32,33^ and 1 was a cross-sectional study.^34^

Descriptive characteristics of the included studies are presented in Table 1. The total number of infants included in the present systematic review was 64 543, with the DDH prevalences’ estimates for Graf type IIb or higher before the age of 3 months ranging from 0.3% to 14.3% within included studies. Only 3 of the included studies were published before 2000, with the majority (14 studies) published in 2015 or after. Considering the screening process, in 9 studies, the screening was universal, and in 6, the screening was selective, leaving 5 studies without defining the screening type. Additionally, in 9 studies, infants undertook US examination in the first week after birth; in 5 studies, US examination was after 4 to 6 weeks after birth; and in 6 studies, US examination was 2 to 3 months after birth.

**Table 1.** Descriptive Characteristics of Included Studies in the Meta-Analysis

Source	Country	Study design	Patients (hips), No.	Prevalence of DDH in study population, %	Time of screening after birth	Screening strategy	Definition of DDH by Graf type
Gardiner et al,^29^ 1990	UK	Case control	164 (328)	3.4	1-2 d	NA (prospective series of preterm infants)	≥IIc
Rosendahl et al,^15^ 1996	Norway	Cohort	3613	3.4	1-2 d	Universal	≥IIc
Falliner et al,^16^ 1999	Germany	Cohort	6548	1.1	1-4 d	Universal	≥IIc
Dogruel et al,^17^ 2008	Turkey	Cohort	3541	4.7	4-6 wk	Universal^a^	≥IIb
Stein-Zamir et al,^30^ 2008	Israel	Case control	205	1.0	10 wk (±7.5 wk)	Selective (only clinical)	≥IIb
De Pellegrin and Moharamzadeh,^31^ 2010	Italy	Case control	1064	4.2	Mean, 64 d (9 wk)	NA	≥IIb
Orak et al,^18^ 2015	Turkey	Cohort	467	0.4	8.9 wk (±1 wk)	Selective^a^	≥IIb
Güler et al,^34^ 2016	Turkey	Cross-sectional	4782 (9564)	0.3	1 mo (4 wk)	Selective	≥IIb
Lange et al,^19^ 2017	Germany	Retrospective cohort	2908 (5816)	1.1^b^	3-10 d	Universal	≥IIc
Vafaee et al,^20^ 2017	Iran	Cohort	1073	4.7	1 wk	Universal	≥IIc
Schams et al,^21^ 2017	Switzerland	Cohort	11 820	2.5	2-5 d	Universal	≥IIc
Onay et al,^22^ 2019	Turkey	Cohort	4415	3.4	Until 12 wk	Universal	≥IIb
Hegde et al,^23^ 2020	Australia	Retrospective observational (cohort)	1110	5.9	6 wk (Corrected age)	NA (only infants with breech)	≥IIb
Demir et al,^32^ 2020	Turkey	Case control	161 (322)	14.3	46 and 188 d (Mean [SD], 80.3 [20.3] d)	NA	≥IIa (Extract ≥IIb)
Treiber et al,^24^ 2021	Slovenia	Cohort	13 404	0.5	1 wk	Universal	≥IIb (Reexamined)
Leonard and Kresch,^25^ 2024	US	Retrospective observational (cohort)	1533	0.4	4-6 wk (Corrected age)	NA (only infants preterm [<35 wk’ gestation])	≥IIb
Koob et al,^33^ 2022	Germany	Case control	660 (1320)	0.9	1 wk	Selective (preterm without any other risk factor for DDH)^a^	≥IIc
Ionescu et al,^26^ 2023	Romania	Retrospective observational (cohort)	3720	4.3	Until 4 mo (16 wk)	Selective (at least 1 risk factor)	≥IIb
Dong et al,^27^ 2024	China	Cohort	3046 (6092)	12.2^b^	Median, 2.2 mo (range, 1.3-3.1 mo) (8 wk)	Selective^a^	≥IIb
Kolovos et al,^28^ 2024	Greece	Retrospective observational (cohort)	309 (618)	2.3^b^	1 wk	Universal^a^	≥IIb

Abbreviations: DDH, developmental dysplasia of the hip; NA, not applicable.

^a^ Not clearly stated.

^b^ Estimated by authors.

Of the 20 included studies, data for meta-analysis were available from 15 studies considering breech presentation, 12 studies for prematurity, 12 for female sex, and 10 studies for family history of DDH, while data for the rest of the risk factors were available in 7 or fewer studies, with 5 studies that included oligohydramnios as a risk factor, 4 studies that included high birth weight (defined as >4000 g), and 6 studies that included low birth weight (defined as <2500 g) (eTable 4 in Supplement 1). Only 1 study had available data about clubfoot (calculated OR, 6.59 [95% CI, 2.68-16.21]), and as a result, it was not possible to conduct a meta-analysis for this proposed risk factor.

eTable 5 in Supplement 1 presents the QUADAS-2 for evaluating risk of bias and applicability in diagnostic accuracy studies. Half of the studies had high risk of bias, 4 had low risk, and 6 were unclear. However, the concern for applicability was mainly low in the majority of studies.

### Meta-Analysis Syntheses

**Figure 2.**
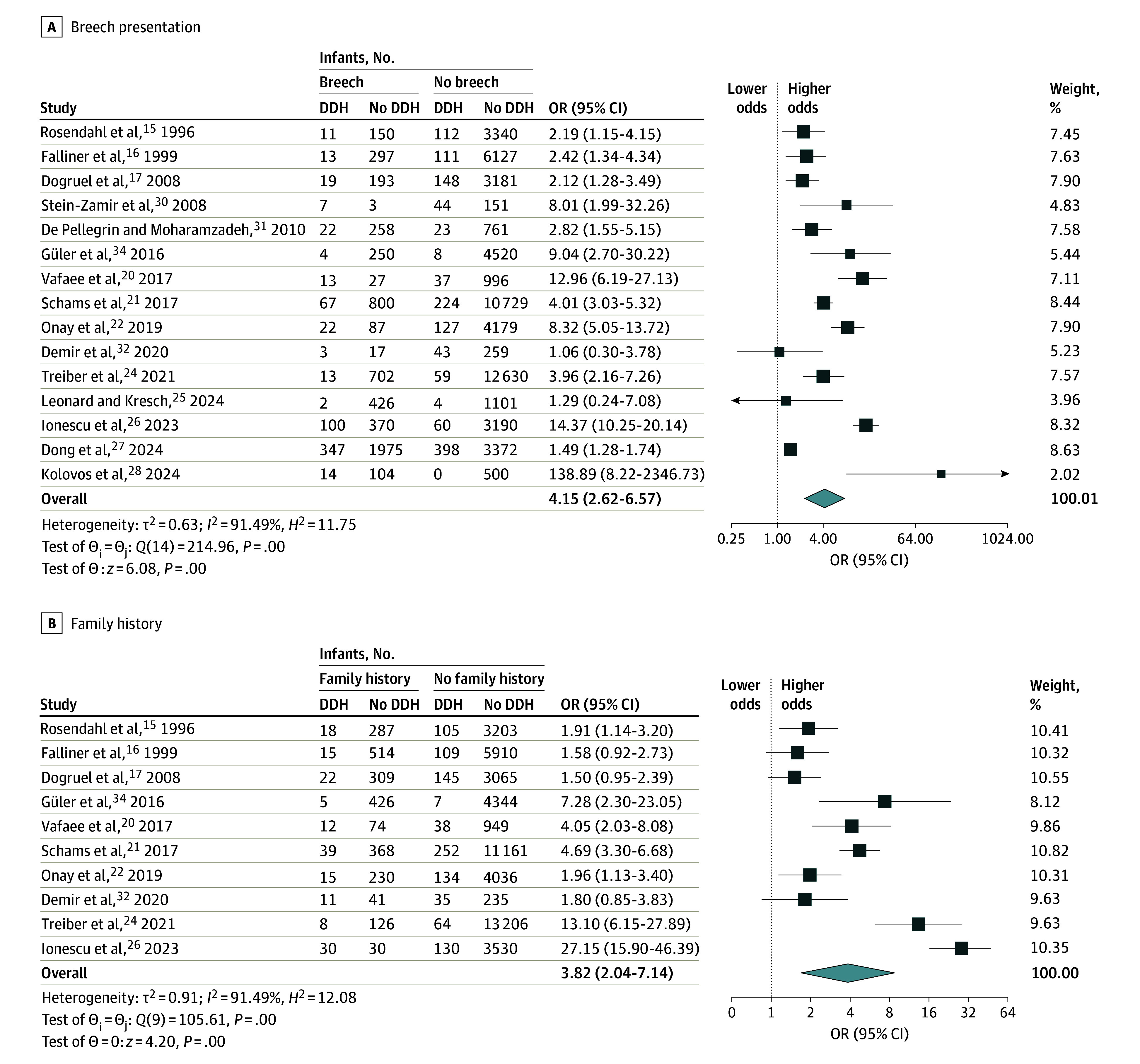
Association of Breech Presentation and Family History With Risk of Developmental Dysplasia of the Hip (DDH) The summary effect sizes were calculated using a random-effects restricted maximum likelihood model. The size of the squares indicates weight; weights may not sum to 100.00% owing to rounding. The horizontal lines represent 95% CIs for the odds ratios (ORs); diamonds represent overall ORs, with outer points indicating 95% CIs. *H*^2^ indicates observed variability; *I*^2^, inconsistency; and *τ*^2^, between-study variance.

**Figure 3.**
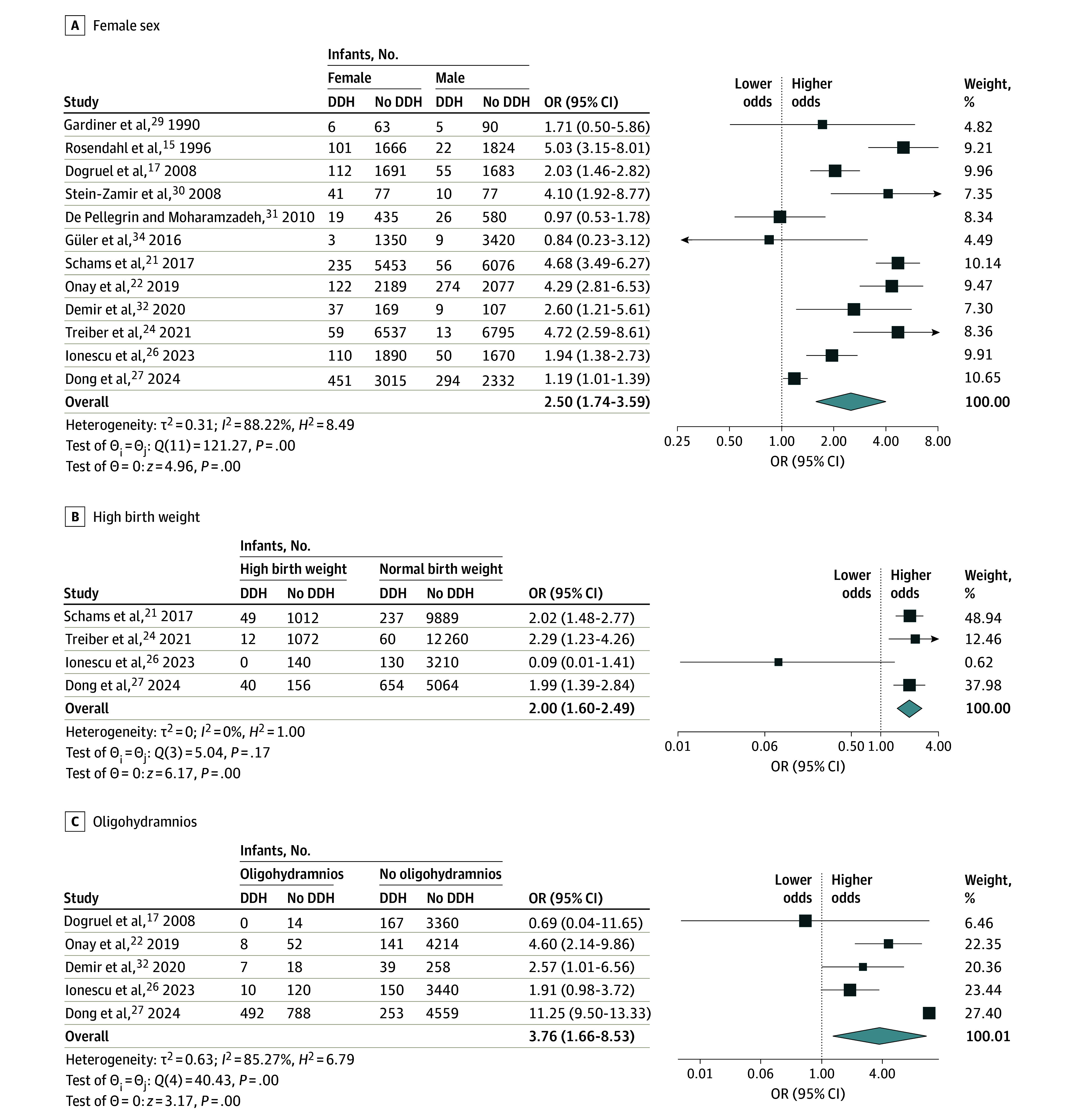
Association of Female Sex, High Birth Weight, and Oligohydramnios With Risk of Developmental Dysplasia of the Hip (DDH) The summary effect sizes were calculated using a random-effects restricted maximum likelihood model. The size of the squares indicates weight; weights may not sum to 100.00% owing to rounding. The horizontal lines represent 95% CIs for the odds ratios (ORs); diamonds represent overall ORs, with outer points indicating 95% CIs. *H*^2^ indicates observed variability; *I*^2^, inconsistency; and *τ*^2^, between-study variance.

The results of the primary meta-analysis syntheses for each risk factor are presented in Table 2. Breech presentation (OR, 4.15 [95% CI, 2.62-6.57]), family history (OR, 3.83 [95% CI, 2.05-7.15]), female sex (OR, 2.50 [95% CI, 1.74-3.59]), high birth weight (OR, 2.00 [95% CI, 1.60-2.49]), and oligohydramnios (OR, 3.76 [95% CI, 1.66-8.53]) were found to be significantly associated with a higher risk of DDH (Figure 2 and Figure 3). However, high heterogeneity was identified (*I*^2^ > 70.00%) for all of the possible risk factors except high birth weight (*I*^2^ > 0%). Cesarian delivery, primiparity (firstborn), low birth weight, multiple births, and prematurity were not found to be associated with a risk of DDH (eFigures 1-5 in Supplement 1).

**Table 2.** Overall Results of the Meta-Analysis for Each Risk Factor

Risk factor	No. of studies	OR (95% CI)	Heterogeneity *I*^2^, %^a^
Breech presentation	15	4.15 (2.62-6.57)	91.49
Cesarean delivery	7	1.21 (0.74-1.98)	89.62
Family history	10	3.83 (2.05-7.15)	91.70
Female sex	12	2.50 (1.74-3.59)	88.22
Firstborn	7	1.62 (0.85-2.74)	93.47
High birth weight	4	2.00 (1.60-2.49)	0
Low birth weight	6	0.84 (0.33-2.16)	93.34
Multiple births	5	0.26 (0.07-1.01)	72.66
Oligohydramnios	5	3.76 (1.66-8.53)	85.27
Prematurity	12	1.03 (0.61-1.73)	84.08

Abbreviation: OR, odds ratio.

^a^ *I*^2^ measures inconsistency.

The sensitivity analysis was performed, and the results are presented in eTable 6 in Supplement 1. The subgroup analyses for every risk factor are presented in eTable 8 in Supplement 1. In most cases, heterogeneity could not be (or was only in small percentages) explained by the sensitivity or subgroup analysis, indicating a significant level of unexplained heterogeneity. For multiple births as a risk factor, the sensitivity analysis accounted for the heterogeneity (OR, 0.14 [95% CI, 0.07-0.29]; *I*^2^ = 0%) when only including studies counting per patient. Additionally, heterogeneity partly accounted for oligohydramnios when only studies counting per patient were included (OR, 2.61 [95% CI, 1.17-5.83]; *I*^2^ = 48.40%). The funnel plots demonstrated symmetric distribution (eFigures 6-9 in Supplement 1), indicating the absence of a significant small study effect (eTable 7 in Supplement 1).

### GRADE Assessment

Using the GRADE guidelines, eTable 9 in Supplement 1 presents the assessments of the certainty in the body of evidence for each risk factor assessed, mainly focusing on the effect estimate. No study could have high certainty since all of the included studies were nonrandomized clinical trials. The certainty was moderate for breech presentation, family history, and multiple births and low for female sex, high birth weight, oligohydramnios, and prematurity. Cesarean delivery, primiparity, and low birth weight had a very low certainty.

## Discussion

Results from this meta-analysis found that breech presentation and family history of DDH are the most significant risk factors for sonographic DDH in infants younger than 3 months, followed by oligohydramnios, female sex, and high birth weight. All of these factors were associated with an increase in the likelihood of an infant developing DDH.

The results of this study partly align with the literature, while also uncovering new insights. Breech presentation and family history were also identified as the main risk factors of DDH, regardless of reference test, in the available meta-analyses on the topic,^6,7,8^ which highlights the genetic predisposition associated with DDH but also the likelihood that limited space in the uterus restricts fetal movement, forcing the hips into adduction. Based on the aforementioned results, the presence of either breech delivery (OR, 4.15 [95% CI, 2.62-6.57]) or a family history of DDH (OR, 3.83 [95% CI, 2.05-7.15]) increases the odds of developing DDH by approximately 4 times.

A similar risk increase was detected for oligohydramnios, which was highlighted in a previous meta-analysis by Chen et al,^8^ in which the OR was 3.93 (95% CI, 1.29-12.01). The DDH risk increase of female sex was found to be lower than previously reported.^7,8^ High birth weight was not included, to our knowledge, in previous meta-analyses on the topic, but our results, as well as other studies, highlight that high birth weight might be pertinent within a multifactorial risk profile for DDH.^35,36^

Our study, in addition to the existing literature, supports that cesarean delivery, low birth weight, and multiple births should not be included as risk factors for selective screening programs, as there is not enough evidence to support their use.^6,7,8^ Prematurity was not associated with DDH, a statement that was also presented in meta-analyses by Ghaseminejad-Raeini et al^37^ and Burkhart et al.^38^

The Newborn and Infant Physical Examination guidelines in the UK specify that a US investigation is warranted for infants with a first-degree family history of DDH, those in breech presentation at or after 36 weeks of pregnancy, and all infants from multiple pregnancies if any of these risk factors are present.^39^ The American Academy of Orthopaedic Surgeons guidelines recommend US screening by the age of 6 to 8 weeks, only for infants with breech presentation, a family history of DDH, or a history of clinical instability, as these are the only risk factors with sufficient evidence to justify screening.^3^ However, based on the results of this meta-analysis, oligohydramnios might infer the same risk increase as breech presentation and family history and thus should be added to the selective screening programs. Future studies should focus on assessing oligohydramnios and high birth weight as risk factors of DDH, rather than prematurity and parity. The only study that we could identify that reported on clubfoot and DDH association estimated the DDH risk increase to be higher than all other risk factors included in the present meta-analysis. However, this estimate of a clubfoot-associated DDH risk increase included wide CIs, signifying the need for more large-scale studies that investigate the effect of clubfoot on DDH risk.

Furthermore, the quality of US examinations, which was included in our quality assessment of the studies, was found to be lacking overall. The quality of hip US examinations varies considerably, with 52% of published studies not presenting correct sonographic images according to the Graf criteria.^40,41^ Incorrect application of the Graf method might lead to variations in the obtained results.^42^ Many of the studies included in the present meta-analysis did not provide a sufficient description of the US methods used. Therefore, establishing a core outcome set for studies reporting on DDH diagnostics might be relevant.

### Strengths and Limitations

A strength of our study is that it is the only meta-analysis to date, to our knowledge, to focus on an age group and a reference test that closely resemble those of existing selective US screening programs: screening infants younger than 3 months and using a Graf US as the reference test. Therefore, we believe the results to be externally valid for all such screening programs. Publication bias was not identified by funnel plots and an Egger test. Additionally, most of the studies were published after 2012, when de Hundt et al^7^ was published, and unlike Chen et al,^8^ we did not limit our analysis to cohort studies. Additionally, sensitivity analysis and publication bias assessment in 3 previous meta-analyses^6,7,8^ were not reported as thoroughly as in the present study.

A notable limitation of the study was the high risk of bias present in half of the included studies. The identified heterogeneity that was in most factors, not possible to be explained by the sensitivity analysis and the subgroup analysis, is also a limitation. A possible explanation could be selective reporting bias of the included studies, as well as different definitions of the risk factors, as not all of the studies provided definitions of the included risk factors. Only including studies published in English language might also be considered a limitation of our study.

## Conclusions

In this meta-analysis, family history of DDH and breech presentation were associated with a significant increase in the risk of sonography-verified DDH in children younger than 3 months. A DDH risk increase of female sex was found to be lower than previously reported. A risk increase was detected for oligohydramnios, which had not been included in many screening programs. This finding may be useful in considering oligohydramnios as an additional risk factor for referral criteria for sonography-verified DDH.

## Notes

Supplement 1. **eTable 1.** Search History November 2023: Hip Dysplasia and Risk Factors **eTable 2.** Excluded Studies With Reason **eTable 3.** Variables Grouped in Order to Be Used for Subgroup Analysis **eTable 4.** Risk Factors Included in Each Study **eTable 5.** QUADAS-2 for Included Studies **eFigure 1.** Forest Plot for Multiple Birth **eFigure 2.** Forest Plot for Low Birth Weight **eFigure 3.** Forest Plot for Cesarean Delivery **eFigure 4.** Forest Plot for Prematurity **eFigure 5.** Forest Plot for Primiparity (Firstborn Child) **eTable 6.** Sensitivity Analysis for Different Risk Factors **eTable 7.** Egger Regression Asymmetry Test for Risk Factor With 10 or More Studies Included in the Meta-Analysis **eFigure 6.** Funnel Plot for Breech Presentation **eFigure 7.** Funnel Plot for Family History **eFigure 8.** Funnel Plot for Female Sex **eFigure 9.** Funnel Plot for Prematurity **eTable 8.** Sensitivity and Subgroup Analysis **eTable 9.** Summary of Findings With GRADE System of Evidence

Supplement 2.
Data Sharing Statement
