# Supplementary material for: Risk Factors for Developmental Dysplasia of the Hip Before 3 Months of Age: A Meta-Analysis
Source: JAMA Netw Open. 2025 Jan 24;8(1):e2456153. doi: 10.1001/jamanetworkopen.2024.56153 (PMC11762239; doi:10.1001/jamanetworkopen.2024.56153)
Supplement: Supplement 1. — eTable 1. Search History November 2023: Hip Dysplasia and Risk Factors eTable 2. Excluded Studies With Reason eTable 3. Variables Grouped in Order to Be Used for Subgroup Analysis eTable 4. Risk Factors Included in Each Study eTable 5. QUADAS-2 for Included Studies eFigure 1. Forest Plot for Multiple Birth eFigure 2. Forest Plot for Low Birth Weight eFigure 3. Forest Plot for Cesarean Delivery eFigure 4. Forest Plot for Prematurity eFigure 5. Forest Plot for Primiparity (Firstborn Child) eTable 6. Sensitivity Analysis for Different Risk Factors eTable 7. Egger Regression Asymmetry Test for Risk Factor With 10 or More Studies Included in the Meta-Analysis eFigure 6. Funnel Plot for Breech Presentation eFigure 7. Funnel Plot for Family History eFigure 8. Funnel Plot for Female Sex eFigure 9. Funnel Plot for Prematurity eTable 8. Sensitivity and Subgroup Analysis eTable 9. Summary of Findings With GRADE System of Evidence [file jamanetwopen-e2456153-s001.pdf]

Supplemental Online Content

Tirta M, Rahbek O, Kold S, Husum HC. Rick factors for developmental dysplasia of the hip before 3 months of age: a meta-analysis. *JAMA Netw Open*. 2025;8(1):e2456153.
doi:10.1001/jamanetworkopen.2024.56153

eTable 1. Search History November 2023: Hip Dysplasia and Risk Factors
eTable 2. Excluded Studies With Reason
eTable 3. Variables Grouped in Order to Be Used for Subgroup Analysis
eTable 4. Risk Factors Included in Each Study
eTable 5. QUADAS-2 for Included Studies
eFigure 1. Forest Plot for Multiple Birth
eFigure 2. Forest Plot for Low Birth Weight
eFigure 3. Forest Plot for Cesarean Delivery
eFigure 4. Forest Plot for Prematurity
eFigure 5. Forest Plot for Primiparity (Firstborn Child)
eTable 6. Sensitivity Analysis for Different Risk Factors
eTable 7. Egger Regression Asymmetry Test for Risk Factor With 10 or More Studies Included in the Meta-Analysis
eFigure 6. Funnel Plot for Breech Presentation
eFigure 7. Funnel Plot for Family History
eFigure 8. Funnel Plot for Female Sex
eFigure 9. Funnel Plot for Prematurity
eTable 8. Sensitivity and Subgroup Analysis
eTable 9. Summary of Findings With GRADE System of Evidence

This supplemental material has been provided by the authors to give readers additional information about their work.

eTable 1. Search History November 2023: Hip Dysplasia and Risk Factors

**Database**	**Result**	**Date**
PubMed National Library of Medicine	2,947	23.11.2023
Embase.com Elsevier	4,397	23.11.2023
Cochrane Library	160	23.11.2023
Total found by database search	7,504	23.11.2023
After duplicate search in Endnote	4,801	23.11.2023

PubMed

Search	Search Details	Results
12	(((("Developmental Dysplasia of the Hip"[MeSH Terms] OR (("dislocat*"[Text Word] OR "dysplasia*"[Text Word] OR "displacement*"[Text Word] OR "instabilit*"[Text Word]) AND ("hip"[Text Word] OR "hips"[Text Word]) AND ("congenital*"[Text Word] OR "developmental*"[Text Word]))) AND ("Risk"[MeSH Terms] OR "Epidemiology"[MeSH Terms] OR "Epidemiology"[MeSH Subheading] OR "Causality"[MeSH Terms] OR "risk*"[Text Word] OR "cause*"[Text Word] OR "occur*"[Text Word] OR "causalit*"[Text Word] OR ("Breech Presentation"[MeSH Terms] OR "Medical History Taking"[MeSH Terms] OR "Parity"[MeSH Terms] OR "pregnancy, multiple"[MeSH Terms] OR "foot deformities, congenital"[MeSH Terms] OR "Birth Weight"[MeSH Terms] OR "Oligohydramnios"[MeSH Terms] OR "Premature Birth"[MeSH Terms] OR "infant, premature"[MeSH Terms] OR "Torticollis"[MeSH Terms]) OR ("Breech"[Text Word] OR "family histor*"[Text Word] OR "Parity"[Text Word] OR "multiple pregnanc*"[Text Word] OR "twin*"[Text Word] OR "foot deformit*"[Text Word] OR "Birth Weight"[Text Word] OR "Oligohydramnios"[Text Word] OR "prematur*"[Text Word] OR "click*"[Text Word]))) NOT "Case Reports"[Publication Type]) NOT ("Animals"[MeSH Terms] NOT "Humans"[MeSH Terms])) AND 1980/01/01:3000/12/31[Date - Publication]	2,947
11	1980/01/01:3000/12/31[Date - Publication]	27,809,411
10	((("Developmental Dysplasia of the Hip"[MeSH Terms] OR (("dislocat*"[Text Word] OR "dysplasia*"[Text Word] OR "displacement*"[Text Word] OR "instabilit*"[Text Word]) AND ("hip"[Text Word] OR "hips"[Text Word]) AND ("congenital*"[Text Word] OR "developmental*"[Text Word]))) AND ("Risk"[MeSH Terms] OR "Epidemiology"[MeSH Terms] OR "Epidemiology"[MeSH Subheading] OR "Causality"[MeSH Terms] OR "risk*"[Text Word] OR "cause*"[Text Word] OR "occur*"[Text Word] OR "causalit*"[Text Word] OR ("Breech Presentation"[MeSH Terms] OR "Medical History Taking"[MeSH Terms] OR "Parity"[MeSH Terms] OR "pregnancy, multiple"[MeSH Terms] OR "foot deformities, congenital"[MeSH Terms] OR "Birth Weight"[MeSH Terms] OR "Oligohydramnios"[MeSH Terms] OR "Premature Birth"[MeSH Terms] OR "infant, premature"[MeSH Terms] OR "Torticollis"[MeSH Terms]) OR ("Breech"[Text Word] OR "family histor*"[Text Word] OR "Parity"[Text Word] OR "multiple pregnanc*"[Text Word] OR "twin*"[Text Word] OR "foot deformit*"[Text Word] OR "Birth Weight"[Text Word] OR "Oligohydramnios"[Text Word] OR "prematur*"[Text Word] OR "click*"[Text Word]))) NOT "Case Reports"[Publication Type]) NOT ("Animals"[MeSH Terms] NOT "Humans"[MeSH Terms])	3,058
9	"Animals"[MeSH Terms] NOT "Humans"[MeSH Terms]	4,941,113
8	(("Developmental Dysplasia of the Hip"[MeSH Terms] OR (("dislocat*"[Text Word] OR "dysplasia*"[Text Word] OR "displacement*"[Text Word] OR "instabilit*"[Text Word]) AND ("hip"[Text Word] OR "hips"[Text Word]) AND ("congenital*"[Text Word] OR "developmental*"[Text Word]))) AND ("Risk"[MeSH Terms] OR "Epidemiology"[MeSH Terms] OR "Epidemiology"[MeSH Subheading] OR "Causality"[MeSH Terms] OR "risk*"[Text Word] OR "cause*"[Text Word] OR "occur*"[Text Word] OR "causalit*"[Text Word] OR ("Breech Presentation"[MeSH Terms] OR "Medical History Taking"[MeSH Terms] OR "Parity"[MeSH Terms] OR "pregnancy, multiple"[MeSH Terms] OR "foot deformities, congenital"[MeSH Terms] OR "Birth Weight"[MeSH Terms] OR "Oligohydramnios"[MeSH Terms] OR "Premature Birth"[MeSH Terms] OR "infant, premature"[MeSH Terms] OR "Torticollis"[MeSH Terms]) OR ("Breech"[Text Word] OR "family histor*"[Text Word] OR "Parity"[Text Word] OR "multiple pregnanc*"[Text Word] OR "twin*"[Text Word] OR "foot deformit*"[Text Word] OR "Birth Weight"[Text Word] OR "Oligohydramnios"[Text Word] OR "prematur*"[Text Word] OR "click*"[Text Word]))) NOT "Case Reports"[Publication Type]	3,134
7	"Case Reports"[Publication Type]	2,238,492
6	("Developmental Dysplasia of the Hip"[MeSH Terms] OR (("dislocat*"[Text Word] OR "dysplasia*"[Text Word] OR "displacement*"[Text Word] OR "instabilit*"[Text Word]) AND ("hip"[Text Word] OR "hips"[Text Word]) AND ("congenital*"[Text Word] OR "developmental*"[Text Word]))) AND ("Risk"[MeSH Terms] OR "Epidemiology"[MeSH Terms] OR "Epidemiology"[MeSH Subheading] OR "Causality"[MeSH Terms] OR "risk*"[Text Word] OR "cause*"[Text Word] OR "occur*"[Text Word] OR "causalit*"[Text Word] OR ("Breech Presentation"[MeSH Terms] OR "Medical History Taking"[MeSH Terms] OR "Parity"[MeSH Terms] OR "pregnancy, multiple"[MeSH Terms] OR "foot deformities, congenital"[MeSH Terms] OR "Birth Weight"[MeSH Terms] OR "Oligohydramnios"[MeSH Terms] OR "Premature Birth"[MeSH Terms] OR "infant, premature"[MeSH Terms] OR "Torticollis"[MeSH Terms]) OR ("Breech"[Text Word] OR "family histor*"[Text Word] OR "Parity"[Text Word] OR "multiple pregnanc*"[Text Word] OR "twin*"[Text Word] OR "foot deformit*"[Text Word] OR "Birth Weight"[Text Word] OR "Oligohydramnios"[Text Word] OR "prematur*"[Text Word] OR "click*"[Text Word]))	3,518
5	"Risk"[MeSH Terms] OR "Epidemiology"[MeSH Terms] OR "Epidemiology"[MeSH Subheading] OR "Causality"[MeSH Terms] OR "risk*"[Text Word] OR "cause*"[Text Word] OR "occur*"[Text Word] OR "causalit*"[Text Word] OR "Breech Presentation"[MeSH Terms] OR "Medical History Taking"[MeSH Terms]	8,359,494

© 2025 Tirta M et al. *JAMA Network Open*.

	OR "Parity"[MeSH Terms] OR "pregnancy, multiple"[MeSH Terms] OR "foot deformities, congenital"[MeSH Terms] OR "Birth Weight"[MeSH Terms] OR "Oligohydramnios"[MeSH Terms] OR "Premature Birth"[MeSH Terms] OR "infant, premature"[MeSH Terms] OR "Torticollis"[MeSH Terms] OR "Breech"[Text Word] OR "family histor*"[Text Word] OR "Parity"[Text Word] OR "multiple pregnanc*"[Text Word] OR "twin*"[Text Word] OR "foot deformit*"[Text Word] OR "Birth Weight"[Text Word] OR "Oligohydramnios"[Text Word] OR "prematur*"[Text Word] OR "click*"[Text Word]	
4	"Breech"[Text Word] OR "family histor*"[Text Word] OR "Parity"[Text Word] OR "multiple pregnanc*"[Text Word] OR "twin*"[Text Word] OR "foot deformit*"[Text Word] OR "Birth Weight"[Text Word] OR "Oligohydramnios"[Text Word] OR "prematur*"[Text Word] OR "click*"[Text Word]	502,174
3	"Breech Presentation"[MeSH Terms] OR "Medical History Taking"[MeSH Terms] OR "Parity"[MeSH Terms] OR "pregnancy, multiple"[MeSH Terms] OR "foot deformities, congenital"[MeSH Terms] OR "Birth Weight"[MeSH Terms] OR "Oligohydramnios"[MeSH Terms] OR "Premature Birth"[MeSH Terms] OR "infant, premature"[MeSH Terms] OR "Torticollis"[MeSH Terms]	183,944
2	"Risk"[MeSH Terms] OR "Epidemiology"[MeSH Terms] OR "Epidemiology"[MeSH Subheading] OR "Causality"[MeSH Terms] OR "risk*"[Text Word] OR "cause*"[Text Word] OR "occur*"[Text Word] OR "causalit*"[Text Word]	8,086,980
1	"Developmental Dysplasia of the Hip"[MeSH Terms] OR (("dislocat*"[Text Word] OR "dysplasia*"[Text Word] OR "displacement*"[Text Word] OR "instabilit*"[Text Word]) AND ("hip"[Text Word] OR "hips"[Text Word]) AND ("congenital*"[Text Word] OR "developmental*"[Text Word]))	10,994

Embase.com

No.	Query	Results
#20	#18 AND #19	4397
#19	[1980-2023]/py	38243684
#18	#17 NOT ([animals]/lim NOT [humans]/lim)	4827
#17	#15 NOT #16	5001
#16	'conference abstract'/it OR 'case report'/exp	7656833
#15	#5 AND #14	6521
#14	#6 OR #7 OR #8 OR #9 OR #10 OR #11 OR #12 OR #13	13877225
#13	breech:ti,ab,kw,de OR 'family histor*':ti,ab,kw,de OR 'parity':ti,ab,kw,de OR 'multiple pregnanc*':ti,ab,kw,de OR twin*:ti,ab,kw,de OR 'foot deformit*':ti,ab,kw,de OR 'birth weight':ti,ab,kw,de OR 'oligohydramnios':ti,ab,kw,de OR prematur*:ti,ab,kw,de OR click*:ti,ab,kw,de	836719
#12	'breech presentation'/exp OR 'family history'/exp OR 'parity'/exp OR 'multiple birth offspring'/exp OR 'foot malformation'/exp OR 'birth weight'/exp OR 'oligohydramnios'/exp OR 'prematurity'/exp OR 'torticollis therapy'/exp	528193
#11	epidemiolog*:ti,ab,kw,de	938854
#10	'epidemiological data'/exp	4621191
#9	'epidemiology'/exp	4599927
#8	'causality'/exp	6612
#7	risk*:ti,ab,kw,de OR cause*:ti,ab,kw,de OR occur*:ti,ab,kw,de OR causalit*:ti,ab,kw,de	10503695
#6	'risk'/exp	3138407
#5	#1 OR #4	16463
#4	#2 AND #3	12323
#3	congenital*:ti,ab,kw,de OR developmental*:ti,ab,kw,de	1087202
#2	(hip* NEAR/3 (dislocat* OR dysplasia* OR displacement* OR instabilit*)):ti,ab,kw,de	27731
#1	'hip dysplasia'/exp	8369

Cochrane Library

ID	Search	Hits
#1	MeSH descriptor: [Developmental Dysplasia of the Hip] explode all trees	130
#2	(hip NEAR/3 (dislocat* OR dyplasia* OR displacement* OR instabilit*)):ti,ab,kw	454
#3	(Congenitial* OR developmental*):ti,ab,kw	8973
#4	#2 AND #3	89
#5	#1 OR #4	160

© 2025 Tirta M et al. *JAMA Network Open*.

eTable 2. Excluded Studies With Reason

1.	Jones DA. Importance of the clicking hip in screening for congenital dislocation of the hip. Lancet (London, England). England; 1989;1(8638):599–601.	No Graf ultrasound
2.	Jones DA, Powell N. Ultrasound and neonatal hip screening. A prospective study of "high risk" babies. J Bone Joint Surg Br. England; 1990;72(3):457–9.	DDH not defined as Graf type ≥IIb
3.	Walter RS, Donaldson JS, Davis CL, Shkolnik A, Binns HJ, Carroll NC, Brouillette RT. Ultrasound screening of high-risk infants: A method to increase early detection of congenital dysplasia of the hip. Am J Dis Child. R.S. Walter, A. I. duPont Institute, Division of General Pediatrics, 1600 Rockland Rd, PO Box 269, Wilmington, DE 19899, United States; 1992;146(2):230–4.	Insufficient data for analysis
4.	Jimenez Jimenez C, Delgado Rodriguez M, Lopez Moratalla M, Sillero Arenas M, Bueno Cavanillas A, Galvez Vargas R. Risk factors of congenital hip dysplasia and maturity deficit of the hip. A case-control study. An Esp Pediatr. M. Delgado Rodriguez, Cat. de Med. Prev./Salud Publica, Facultad de Medicina, Universidad de Cantabria, Avda. Cardenal Herrera Oria, s/n, 39011 Santander, Spain; 1995;43(3):191–6.	Full text in non-English
5.	Stoffelen D, Urlus M, Molenaers G, Fabry G. Ultrasound, radiographs, and clinical symptoms in developmental dislocation of the hip: a study of 170 patients. J Pediatr Orthop B. United States; 1995;4(2):194–9.	No comparison group
6.	Holen KJ, Tegnander A, Terjesen T, Johansen OJ, Eik-Nes SH. Ultrasonographic evaluation of breech presentation as a risk factor for hip dysplasia. Acta Paediatr. Norway; 1996;85(2):225–9.	No Graf ultrasound
7.	Boere-Boonekamp MM, Kerkhoff THM, Schuil PB, Zielhuis GA. Early detection of developmental dysplasia of the hip in the Netherlands: The validity of a standardized assessment protocol in infants. Am J Public Health. M.M. Boere-Boonekamp, Centre for Health Care Research, University of Twen, PO Box 217, 7500 AE Enschede, Netherlands; 1998;88(2):285–8.	Population too old (> 3 months at US examination)
8.	Rühmann O, Konermann W, Lazović D, Vitek L, Bouklas P. [Ultrasound neonatal screening: the effect of anamnestic risk factors on hip dysplasia]. Z Orthop Ihre Grenzgeb. Germany; 1998;136(6):492–500.	Full text in non-English
9.	Von Deimling U, Brähler JM, Niesen M, Wagner UA, Walpert J. Relationship of birth weight and congenital dysplasia of hip (CDH) in newborn children. Klin Padiatr. U. Von Deimling, Leiter Sekt. Wirbelsaulenchirurgie, Ev. Krankenhaus Lutherhaus, Hellweg 100, D-45276 Essen, Germany; 1998;210(3):115–9.	Full text in non-English
10.	Rühmann O, Lazović D, Bouklas P, Rössig S. Ultrasound screening of neonatal hip. Is twin pregnancy a risk factor for congenital dysplasia? Ultraschall der Medizin. O. Ruhmann, Orthopadische Klinik, Medizinische Hochschule Hannover, Heimchenstrasse 1-7, D-30625 Hannover, Germany; 1998;19(2):64–9.	Full text in non-English
11.	Rühmann O, Lazović D, Bouklas P, Gossé F, Franke J. Ultrasound screening of neonatal hip. Correlation of anamnestic risk factors and congenital dysplasia. Klin Padiatr. O. Ruhmann, Orthopadische Klinik, Medizinischen Hochschule Hannover, Heimchenstr. 1-7, D-30625 Hannover, Germany; 1999;211(3):141–8.	DDH not defined as Graf type ≥IIb
12.	Rühmann O, Lazović D, Bouklas P, Schmolke S, Flamme CH. Ultrasound examination of neonatal hip: correlation of twin pregnancy and congenital dysplasia. Twin Res. Australia; 2000;3(1):7–11.	DDH not defined as Graf type ≥IIb
13.	Abela M, Benson MKD. Risk factors in developmental dysplasia of the hip. HIP Int. M.K.D. Benson, Nuffield Orthopaedic Centre, Windmill Road, Headington, Oxford OX3 7LD, United Kingdom; 2001;11(3):127–33.	No Graf ultrasound
14.	Omeroğlu H, Koparal S, Ömeroğlu H, Koparal S. The role of clinical examination and risk factors in the diagnosis of developmental dysplasia of the hip: A prospective study in 188 referred young infants. Arch Orthop Trauma Surg. H. Ömeroğlu, Turgut Reis Caddesi 54/8, 06570 Ankara, Turkey, Germany; 2001;121(1–2):7–11.	Population too old (> 3 months at US examination)
15.	Paton RW, Hossain S, Eccles K. Eight-year prospective targeted ultrasound screening program for instability and at-risk hip joints in developmental dysplasia of the hip. J Pediatr Orthop. United States; 2002;22(3):338–41.	DDH not defined as Graf type ≥IIb
16.	Giannakopoulou C, Aligizakis A, Korakaki E, Velivasakis E, Hatzidaki E, Manoura A, Bakataki A, Hadjipavlou A. Erratum: Neonatal screening for developmental dysplasia of the hip on the maternity wards in Crete, Greece. Correlation to risk factors (Clinical and Experimental Obstetrics and Gynecology (2002) 29:2 (148)). Clin Exp Obstet Gynecol. University of Creete, Crete, Greece; 2002;29(3):155.	DDH not defined as Graf type ≥IIb

© 2025 Tirta M et al. *JAMA Network Open*.

17.	Bache CE, Clegg J, Herron M. Risk factors for developmental dysplasia of the hip: Ultrasonographic findings in the neonatal period. J Pediatr Orthop Part B. C.E. Bache, 109 Wood Lane, Harborne, Birmingham B17 9AY, United Kingdom; 2002;11(3):212–8.	DDH not defined as Graf type ≥IIb
18.	Kremli MK, Alshahid AH, Khoshhal KI, Zamzam MM. The pattern of developmental dysplasia of the hip. Saudi Med J. Saudi Arabia; 2003;24(10):1118–20.	No Graf ultrasound
19.	Şahin F, Aktürk A, Beyazova U, Çakir B, Boyunağa Ö, Tezcan S, Bölükbaşi S, Kanatli U. Screening for developmental dysplasia of the hip: Results of a 7-year follow-up study. Pediatr Int. F. Şahin, Gazi University, Faculty of Medicine, Department of Pediatrics, Oyak Sitesi 7. Blok No: 7, 06550 Çankaya, Ankara, Turkey; 2004;46(2):162–6.	DDH not defined as Graf type ≥IIb
20.	Paton RW, Hinduja K, Thomas CD, Hinduja " K. The significance of at-risk factors in ultrasound surveillance of developmental dysplasia of the hip A TEN-YEAR PROSPECTIVE STUDY. J BONE Jt Surg J Bone Jt Surg [Br]. 2005;87(Ci):1264–6.	DDH not defined as Graf type ≥IIb
21.	Vukelić-Šarunić A, Tomašić-Martinis E, Prpić I, Krajina R, Zaputović S. Breech praesentation - Risk factor for developmental dysplasia of the hip. Gynaecol Perinatol Suppl. Klinika za Ginekologiju i Porodništvo, Klinički Bolnički Centar Rijeka, Odsjek Neonatologije,; 2005;14(2):94–7.	Full text in non-English
22.	Tuncay IC, Karaeminogullari O, Demirörs H, Tandogan NR. Is prematurity important in ultrasonographic hip typing? J Pediatr Orthop B. United States; 2005;14(3):168–71.	Missing DDH definition
23.	Lowry CA, Donoghue VB, Murphy JF. Auditing hip ultrasound screening of infants at increased risk of developmental dysplasia of the hip. Arch Dis Child. 2005;90(6):579–81.	Missing DDH definition
24.	Lowry CA, Donoghue VB, O'Herlihy C, Murphy JF. Elective Caesarean section is associted with a reduction in developmental dysplasia of the hip in term breech infants. J Bone Jt Surg - Ser B. C.A. Lowry, Department of Neonatology, National Maternity Hospital, Holles Street, Dublin 2, Ireland; 2005;87(7):984–5.	Missing DDH definition
25.	Akman A, Korkmaz A, Aksoy MC, Yazici M, Yurdakök M, Tekinalp G. Evaluation of risk factors in developmental dysplasia of the hip: results of infantile hip ultrasonography. Turk J Pediatr. Turkey; 2007;49(3):290–4.	Missing information on risk factor status
26.	De Pellegrin M, Moharamzadeh D, Fraschini G. Early diagnosis and treatment of DDH: A sonographic approach. HIP Int. M. De Pellegrin, Services of Paediatric Orthopaedic Surgery, Department of Othopaedics and Traumatology, Università Vita-Salute IRCCS San Raffaele, Via Olgettina 60, 20132 Milan, Italy; 2007;17(SUPPL. 5):S15–21.	DDH not defined as Graf type ≥IIb
27.	Akgün S, Bakar C, Budakoglu II, Tuncay C, Cemil T. Is clinical examination reliable in diagnosis of developmental dysplasia of the hip? Gazi Med J. S. Akgün, Başkent University, Faculty of Medicine, Department of Public Health, 12 Street 7/6, 06490 Bahçelievler, Ankara, Turkey; 2008;19(2):56–9.	DDH not defined as Graf type ≥IIb
28.	Treiber M, Tomazic T, Tekauc-Golob A, Zolger J, Korpar B, Burja S, Takac I, Sikosek A. Ultrasound screening for developmental dysplasia of the hip in the newborn: a population-based study in the Maribor region, 1997-2005. Wien Klin Wochenschr. Austria; 2008;120(1–2):31–6.	Missing information on risk factor status
29.	Sionek A, Czubak J, Kornacka M, Grabowski B. Evaluation of risk factors in developmental dysplasia of the hip in children from multiple pregnancies: results of hip ultrasonography using Graf's method. Ortop Traumatol Rehabil. Poland; 2008;10(2):115–30.	DDH not defined as Graf type ≥IIb
30.	Simić S, Vukasinović Z, Samardzić J, Pejcić I, Lukavac-Tesin M, Spasovski D, Bozinović-Prekajski N. Does the gestation age of newborn babies influence the ultrasonic assessment of hip condition? Srp Arh Celok Lek. Serbia; 2009;137(7–8):402–8.	DDH not defined as Graf type ≥IIb<br><br>Population too old (> 3 months at US examination)
31.	Stevenson DA, Mineau G, Kerber RA, Viskochil DH, Schaefer C, Roach JW. Familial predisposition to developmental dysplasia of the hip. J Pediatr Orthop. United States; 2009;29(5):463–6.	No information on US results
32.	Fazlagic S, Grubor P, Fazlagic S. Risk factors for development of hip disorder among newborn babies in Tesanj region. Med Arh. Bosnia and Herzegovina; 2010;64(6):339–44.	DDH not defined as Graf type ≥IIb
33.	Šegregur J, Koši-Šantić K, Hrgović Z. Perinatal factors and developmental displacement of the hip. Gynaecol Perinatol. J. Šegregur, Odjel Za Ženske Bolesti i Porodništvo, Opća Bolnica Virovitica, Gajeva 21, 33 000 Virovitica, Croatia; 2011;20(1):1–5.	Full text in non-English
34.	Yazgan H, Keleş E, Gebeşçi A, Demirdöven M, Baştürk B, Etlik Ö. Our four-years results of developmental hip dysplasia screening program in newborns. Turkiye Klin Pediatr. H. Yazgan, Private Sema Hospital, Clinic of Pediatrics, Istanbul, Turkey; 2012;21(1):7–10.	Missing outcome information

© 2025 Tirta M et al. *JAMA Network Open*.

35.	Šantić KK, Vrdoljak O, Vrdoljak J, Hrgović Z. Ultrasonographic screening for developmental displacement of the hip at Department of Pediatrics, Virovitica General Hospital. Paediatr Croat. K.K. Šantić, Odjel za Dječje Bolesti, Opća Bolnica Virovitica, Gajeva 21, 33000 Virovitica, Croatia; 2012;56(1):15–9.	Full text in non-English
36.	De Hundt M, Vlemmix F, Bais JMJ, Hutton EK, De Groot CJ, Mol BWJ, Kok M. Risk factors for developmental dysplasia of the hip: A meta-analysis. Eur J Obstet Gynecol Reprod Biol. M. De Hundt, Department of Obstetrics and Gynaecology, Medical Centre Alkmaar, Alkmaar, Netherlands; 2012;165(1):8–17.	Systematic review
37.	Grubor P, Grubor M, Domuzin M, Golubović I. Value of the first examination for developmental dysplasia of the hip - our experiences. Acta Fac Medicae Naissensis. P. Grubor, Traumatology Clinic, Clinical Centre Banja Luka, Bosnia and Herzegovina; 2012;29(3):125–31.	Missing outcome information
38.	Ortiz-Neira CL, Paolucci EO, Donnon T. A meta-analysis of common risk factors associated with the diagnosis of developmental dysplasia of the hip in newborns. Eur J Radiol. Ireland; 2012;81(3):e344-51.	Systematic review
39.	Panagiotopoulou N, Bitar K, Hart WJ. The association between mode of delivery and developmental dysplasia of the hip in breech infants: a systematic review of 9 cohort studies. Acta Orthop Belg. Belgium; 2012;78(6):697–702.	Systematic review
40.	Talbot CL, Paton RW. Screening of selected risk factors in developmental dysplasia of the hip: An observational study. Arch Dis Child. C.L. Talbot, Orthopaedic Department, East Lancashire Hospitals NHS Trust, Royal Blackburn Hospital, Haslingden Road, Blackburn BB2 3HH, United Kingdom; 2013;98(9):692–6.	DDH not defined as Graf type ≥IIb
41.	Barr L V., Rehm A. Should all twins and multiple births undergo ultrasound examination for developmental dysplasia of the hip?: A retrospective study of 990 multiple births. Bone Joint J. 2013;95-B(1):132–4.	Missing outcome information
42.	Li L, Sun K, Zhang L, Zhao Q, Cheng X, Dang Y. Heritability and sibling recurrent risk of developmental dysplasia of the hip in Chinese population. Eur J Clin Invest. England; 2013;43(6):589–94.	No Graf ultrasound
43.	Quan T, Kent AL, Carlisle H. Breech preterm infants are at risk of developmental dysplasia of the hip. J Paediatr Child Health. Australia; 2013;49(8):658–63.	No Graf ultrasound
44.	Mahan ST, Yazdy MM, Kasser JR, Werler MM. Is it worthwhile to routinely ultrasound screen children with idiopathic clubfoot for hip dysplasia? J Pediatr Orthop. 2013;33(8):847–51.	Missing outcome information
45.	Duramaz A, Peker G, Arslan L, Bilgili MG, Erçin E, Kural C. Hip ultrasonography in the diagnosis of developmental dysplasia of the hip: Bakırköy experience. Haseki Tip Bul. A. Duramaz, Özel Medicana Hastanesi, Ortopedi ve Travmatoloji Kliniği, İstanbul, Turkey; 2014;52(4):262–7.	Full text in non-English
46.	Paton RW, Choudry QA, Jugdey R, Hughes S. Is congenital talipes equinovarus a risk factor for pathological dysplasia of the hip? : a 21-year prospective, longitudinal observational study. Bone Joint J. England; 2014;96-B(11):1553–5.	No comparison group
47.	LeBa T-B, Carmichael KD, Patton AG, Morris RP, Swischuk LE. Ultrasound for Infants at Risk for Developmental Dysplasia of the Hip. Orthopedics. United States; 2015;38(8):e722-6.	No Graf ultrasound
48.	Woodacre T, Ball T, Cox P. Epidemiology of developmental dysplasia of the hip within the UK: refining the risk factors. J Child Orthop. 2016;10(6).	Missing raw numbers for outcome analysis
49.	Colta RC, Stoicanescu C, Nicolae M, Oros S, Burnei G. Hip dysplasia screening - epidemiological data from Valcea County. J Med Life. 2016;9(1).	Missing raw numbers for outcome analysis
50.	Kolb A, Schweiger N, Mailath-Pokorny M, Kaider A, Hobusch G, Chiari C, Windhager R. Low incidence of early developmental dysplasia of the hip in universal ultrasonographic screening of newborns: analysis and evaluation of risk factors. Int Orthop. Germany; 2016;40(1):123–7.	Missing raw numbers for outcome analysis
51.	Sağlam N, Kaya İ, Sungur İ, Sarıyılmaz K, Sağlam NÖ, Türkmen İ. Risk factors for developmental dysplasia of the hip: Results from 1025 neonates. Nobel Med. İ. Türkmen, Istanbul Beykoz State Hospital, Saip Molla Cd. Kısayol sok. No:1, Beykoz, Istanbul, Turkey; 2017;13(2):76–9.	Missing DDH definition
52.	Pollet V, Percy V, Prior HJ. Relative Risk and Incidence for Developmental Dysplasia of the Hip. J Pediatr. United States; 2017;181:202–7.	Missing raw numbers for outcome analysis
53.	Kural B, Devecioğlu Karapınar E, Yılmazbaş P, Eren T, Gökçay G. Risk Factor Assessment and a Ten-Year Experience of DDH Screening in a Well-Child Population. Biomed Res Int. 2019;2019:7213681.	DDH not defined as Graf type ≥IIb

© 2025 Tirta M et al. *JAMA Network Open*.

		Population too old (> 3 months at US examination)
54.	Alrowaili MG, Alrowali HBH, Alrowaly SHR. The incidence of neonatal developmental dysplasia of the hip in Arar, Saudi Arabia. Ann Clin Anal Med. M.G. Alrowaili, Department of Surgery, Faculty of Medicine, Northern Border University, P.O. Box 1321, Arar, Saudi Arabia; 2019;11(3):179–82.	Missing DDH definition
55.	Mureşan S, Mărginean MO, Voidăzan S, Vlasa I, Sîntean I. Musculoskeletal ultrasound: a useful tool for diagnosis of hip developmental dysplasia: One single-center experience. Medicine (Baltimore). 2019;98(2):e14081.	DDH not defined as Graf type ≥IIb
56.	Ömeroğlu H, Akceylan A, Köse N. Associations between risk factors and developmental dysplasia of the hip and ultrasonographic hip type: a retrospective case control study. J Child Orthop. 2019;13(2):161–6.	DDH not defined as Graf type ≥IIb
57.	D'Alessandro M, Dow K. Investigating the need for routine ultrasound screening to detect developmental dysplasia of the hip in infants born with breech presentation. Paediatr Child Health. 2019;24(2):e88–93.	DDH not defined as Graf type ≥IIb
58.	Barbosa R de O, Albernaz EP. Profile of Patients Diagnosed with Developmental Dysplasia of the Hip. Rev Bras Ortop. 2019;54(5):497–502.	Incorrect study design – questionnaire
59.	Manoukian D, Rehm A. Oligohydramnios: should it be considered a risk factor for developmental dysplasia of the hip? J Pediatr Orthop B. United States; 2019;28(5):442–5.	Missing raw numbers for outcome analysis
60.	Roposch A, Protopapa E, Malaga-Shaw O, Gelfer Y, Humphries P, Ridout D, Wedge JH. Predicting developmental dysplasia of the hip in at-risk newborns. BMC Musculoskelet Disord. A. Roposch, Institute of Child Health, University College London, 30 Guildford St, London, United Kingdom; 2020;21(1).	DDH not defined as Graf type ≥IIb
61.	Håberg, Foss OA, Lian B, Holen KJ. Is foot deformity associated with developmental dysplasia of the hip? results after examination of 60,844 newborns. Bone Jt J. 2020;102(11):1582–6.	DDH not defined as Graf type ≥IIb
62.	Håberg, Foss OA, Lian B, Holen KJ. Is foot deformity associated with developmental dysplasia of the hip? results after examination of 60,844 newborns. Bone Jt J. 2020;102(11):1582–6.	DDH not defined as Graf type ≥IIb
63.	Wang Y, Wang J, Sheng C, Chen Z, Li J. Incidence and epidemiological characteristics of developmental dysplasia of the hip in Yangzhou. Chinese J Pediatr Surg. J. Wang, Department of Pediatric Orthopedics, Yangzhou Maternal and Child Health Care Hospital, Affiliated Hospital of Medical College of Yangzhou University, Yangzhou, China; 2021;42(8):727–31.	Full text in non-English
64.	Wang Y, Wang J, Sheng C, Chen Z, Li J. Incidence and epidemiological characteristics of developmental dysplasia of the hip in Yangzhou. Chinese J Pediatr Surg. J. Wang, Department of Pediatric Orthopedics, Yangzhou Maternal and Child Health Care Hospital, Affiliated Hospital of Medical College of Yangzhou University, Yangzhou, China; 2021;42(8):727–31.	DDH not defined as Graf type ≥IIb
65.	Angsanuntsukh C, Patathong T, Klaewkasikum K, Jungtheerapanich W, Saisongcroh T, Mulpruek P, Woratanarat P. Factors for selective ultrasound screening in newborns with developmental dysplasia of the hip (DDH). Front Surg. Switzerland; 2022;9:1038066.	No Graf ultrasound
66.	Oh EJ, Min JJ, Kwon S-S, Kim SB, Choi CW, Jung YH, Oh KJ, Park JY, Park MS. Breech Presentation in Twins as a Risk Factor for Developmental Dysplasia of the Hip. J Pediatr Orthop. 2022;42(1):e55–8.	Incorrect study population
67.	Norlén S, Faergemann C. Developmental dysplasia of the hip in infants referred for a combined pediatric orthopedic and radiologic examination. A prospective cohort study. J Orthop. 2022;32:109–14.	DDH not defined as Graf type ≥IIb
68.	Xu N, Xia B, Tao H, Sun K, Liu Q, Chen W, Wang D, Gao H, Guo Y, Liu Y, Gao J, Teng J, Li T, He Q, Wu Z. Epidemiological investigation and ultrasonic diagnosis of developmental dysplasia of the hip in Chinese infants: A large multi-center cohort study. Medicine (Baltimore). United States; 2022;101(2):e28320.	Missing DDH definition
69.	Ziegler CM, Ertl KM, Delius M, Foerster KM, Crispin A, Wagner F, Heimkes B. Clinical examination and patients' history are not suitable for neonatal hip screening. J Child Orthop. England; 2022;16(1):19–26.	Missing raw numbers for outcome analysis
70.	Hsu K-H, Chang W-C, Feng C-K, Su Y-P. Implementing the AAOS Guidelines for Screening of Developmental Dysplasia of the Hip before the Age of 6 Months in Taiwan. J Pediatr Orthop. K.-H. Hsu, Department of Orthopaedics and Traumatology, Taipei Veterans General Hospital, Taipei, Taiwan; 2023;43(6):E416–20.	Missing raw numbers for outcome analysis

© 2025 Tirta M et al. *JAMA Network Open*.

71.	Chatziravdeli V, Kazas C, Metaxiotis D, Chatziioannidis I, Mitsiakos G, Diamanti E. Sonographic Hip Angles in Relation to Gestational Age of Neonates. A Prospective, Cohort in the Population of Northern Greece. Arch bone Jt Surg. Iran; 2023;11(3):197–205.	Missing raw numbers for outcome analysis
72.	Osman A, Jackson K, Conroy S, Seguin J, Slaughter JL. The Risk of Developmental Dysplasia of the Hip in Premature Infants with Breech Presentation at Birth. Am J Perinatol. A. Osman, Department of Pediatrics, Nationwide Children's Hospital, The Ohio State University, College of Medicine, 700 Children's Dr. Faculty Office Building, FB6353, Columbus, OH, United States; 2023;	DDH not defined as Graf type ≥IIb
73.	Burkhart RJ, McNassor R, Acuña AJ, Kamath AF. Is prematurity a risk factor for developmental dysplasia of the hip? A systematic review and meta-analysis. J Pediatr Orthop Part B. A.F. Kamath, Center for Hip Preservation, Orthopaedic and Rheumatologic Institute, Cleveland Clinic Foundation, 9500 Euclid Ave, Mail code A41, Cleveland, OH, United States; 2023;32(4):305–11.	Systematic review
74.	Ghaseminejad-Raeini A, Shahbazi P, Roozbahani G, Sharafi A, Shafiei SH, Fallah Y, Baghdadi S. Preterm birth does not increase the risk of developmental dysplasia of the Hip: a systematic review and meta-analysis. BMC Pediatr. S. Baghdadi, Division of Orthopaedics, The Children's Hospital of Philadelphia, Philadelphia, PA, United States; 2023;23(1).	Systematic review
75.	Poacher AT, Froud JLJ, Caterson J, Crook DL, Ramage G, Marsh L, Poacher G, Carpenter EC. The cost effectiveness of potential risk factors for developmental dysplasia of the hip within a national screening programme. Bone Jt open. England; 2023;4(4):234–40.	Missing raw numbers for outcome analysis
76.	Candussi I-L, Ene D, Bușilă C, Mihailov R, Sârbu I, Lungu CN, Ciongradi CI. The Influence of Risk Factors in the Early Detection of Developmental Dysplasia of the Hip in a Country with Limited Material Resources. Healthc (Basel, Switzerland). Switzerland; 2023;11(17).	Missing raw numbers for outcome analysis
77.	Fox AE, Paton RW. The relationship between mode of delivery and developmental dysplasia of the hip in breech infants: a four-year prospective cohort study. J Bone Joint Surg Br. England; 2010;92(12):1695–9.	DDH not defined as Graf type ≥IIb
78.	Dong T, Nie F, Wei J, Feng J, Liu W, Yang Y. High-frequency ultrasound in diagnosis of infant developmental dysplasia of hip. Chinese J Med Imaging Technol. F. Nie, Department of Ultrasound, The Second Hospital of Lanzhou University, Lanzhou, China; 2018;34(7):1076–80.	Full text in non-English
79.	Tan SHS, Wong KL, Hui JH. Incorporating risk factors in the development of the screening programme for developmental dysplasia of the hips. J Pediatr Orthop B. United States; 2019;28(2):111–4.	DDH not defined as Graf type ≥IIb
80.	Buonsenso D, Curatola A, Lazzareschi I, Panza G, Morello R, Marrocco R, Valentini P, Cota F, Rendeli C. Developmental dysplasia of the hip: real world data from a retrospective analysis to evaluate the effectiveness of universal screening. J Ultrasound. 2021;24(4):403–10.	DDH not defined as Graf type ≥IIb
81.	Kosar P, Ergun E, Gökharman FD, Turgut AT, Kosar U. Follow-up sonographic results for Graf type 2A hips: association with risk factors for developmental dysplasia of the hip and instability. J ultrasound Med Off J Am Inst Ultrasound Med. England; 2011;30(5):677–83.	DDH not defined as Graf type ≥IIb
82.	Jeon GW, Choo HJ, Kwon YU. Risk factors and screening timing for developmental dysplasia of the hip in preterm infants. Clin Exp Pediatr. G.W. Jeon, Department of Pediatrics, Inha University Hospital, Inha University College of Medicine, 27 Inhang-ro, Jung-gu, Incheon, South Korea; 2022;65(5):262–8.	DDH not defined as Graf type ≥IIb
83.	Xiao H, Tang Y, Su Y. Risk factors of developmental dysplasia of the hip in a single clinical center. Sci Rep. England; 2022;12(1):19461	DDH not defined as Graf type ≥IIb
84.	Pulik Ł, Płoszka K, Romaniuk K, Sibilska A, Jedynak A, Tołwiński I, Kumięga P, Wojtyński P, Łęgosz P. Impact of Multiple Factors on the Incidence of Developmental Dysplasia of the Hip: Risk Assessment Tool. Medicina (Kaunas). Switzerland; 2022;58(9).	DDH not defined as Graf type ≥IIb
85.	Zhu D, Zhu H. Incidence and Epidemiological Characters of Developmental Dysplasia of the Hip in Lianyungang: Based on Ultrasound Screening: A Retrospective Study. Int J Gen Med. D. Zhu, Department of Pediatric Orthopedics, The First People's Hospital of Lianyungang, Lianyungang, China; 2022;15:8547–55.	DDH not defined as Graf type ≥IIb
86.	Alshehri F, Almalki Y. Developmental dysplasia of the hip in infants younger than six months: Ultrasonographic assessment in relation with risk factors. Int J Health Sci (Qassim). Saudi Arabia; 2023;17(2):37–45.	Population too old. (> 3 months at US examination)

eTable 3. Variables Grouped in Order to Be Used for Subgroup Analysis

Study	Study design	Time of screening	Screening	Definition cases	Risk of Bias*
Gardiner 1990	Case-Control	1 week	N/a	≥ IIc	High
Rosendahl 1996	Cohort	1 week	Universal	≥ IIc	Low
Falliner 1999	Cohort	1 week	Universal	≥ IIc	Unclear
Dogruel 2008	Cohort	4-6 weeks	Universal	≥ IIb	High
Stein-Zamir 2008	Case-Control	2-3 months	Selective	≥ IIb	High
De-Pellegrin 2010	Case-Control	4-6 weeks	N/a	≥ IIb	High
Orak 2015	Cohort	2-3 months	Selective	≥ IIb	High
Güler 2016	Cross-sectional	4-6 weeks	Selective	≥ IIb	High
Lange 2017	Cohort	1 week	Universal	≥ IIc	Unclear
Vafaee 2017	Cohort	1 week	Universal	≥ IIc	Unclear
Schams 2017	Cohort	1 week	Universal	≥ IIc	Low
Onay 2019	Cohort	2-3 months	Universal	≥ IIb	Unclear
Hegde 2020	Cohort	4-6 weeks	N/a	≥ IIb	High
Demir 2020	Case-Control	2-3 months	N/a	≥ IIb	High
Treiber 2021	Cohort	1 week	Universal	≥ IIb	Unclear
Leonard 2022	Cohort	4-6 weeks	N/a	≥ IIb	Unclear
Koob 2022	Case-Control	1 week	Selective	≥ IIc	High
Ionescu 2023	Cohort	2-3 months	Selective	≥ IIb	High
Dong 2023	Cohort	2-3 months	Selective	≥ IIb	Low
Kolovos 2023	Cohort	1 week	Universal	≥ IIb	Low

*Summarized by Robvis tool

© 2025 Tirta M et al. *JAMA Network Open*.

	**Female sex**	**Breech presentation**	**Family history**	**Prematurity**	**Primatirity**	**Low birthweight**	**High birthweight**	**Multiple birth**	**Type of Birth**	**Oligohydramnios**	**Clubfoot**
**Gardiner 1990**	X			X							
**Rosendahl 1996**	X	X	X								
**Falliner 1999**		X	X								
**Dogruel 2008**	X	X	X	X	X	X			X	X	
**Stein-Zamir 2008**	X	X		X	X	X			X		
**De-Pellegrin 2010**	X	X						X			
**Orak 2015**				X							
**Güler 2016**	X	X	X		X						
**Lange 2017**				X							
**Vafaee 2017**		X	X								
**Schams 2017**	X	X	X	X		X	X	X			
**Onay 2019**	X	X	X	X		X		X		X	X
**Hegde 2020**				X					X		
**Demir 2020**	X	X	X	X	X			X	X	X	
**Treiber 2021**	X	X	X		X		X		X		
**Leonard 2022**		X									
**Koob 2022**				X							
**Ionescu 2023**	X	X	X	X	X	X	X	X	X	X	
**Dong 2023**	X	X		X	X	X	X		X	X	
**Kolovos 2023**		X									
**Total number of studies**	12	15	10	12	7	6	4	5	7	5	1

© 2025 Tirta M et al. *JAMA Network Open*.

eTable 4. Risk Factors Included in Each Study

Study	Risk of bias				Applicability concerns		
	Patient selection	Index test	Reference Standard	Flow and timing	Patient selection	Index test	Reference standard
Gardiner 1990	2	3	2	2	1	1	1
Rosendahl 1996	1	1	1	1	1	1	1
Falliner 1999	3	1	3	1	1	1	1
Dogruel 2008	2	3	3	2	1	1	1
Stein-Zamir 2008	2	2	2	2	2	1	2
De-Pellegrin 2010	1	1	3	2	1	1	1
Orak 2015	1	1	3	2	1	1	1
Güler 2016	1	2	3	1	1	1	1
Lange 2017	1	3	3	1	1	1	1
Vafaee 2017	3	3	3	3	3	1	1
Schams 2017	1	1	1	1	1	1	1
Onay 2019	3	1	3	1	1	1	1
Hegde 2020	2	3	3	3	1	1	1
Demir 2020	2	2	3	2	2	2	1
Treiber 2021	3	1	3	3	1	1	1
Leonard 2022	3	1	3	1	3	3	1
Koob 2022	2	3	3	1	1	1	1
Ionescu 2023	3	1	1	2	2	1	1
Dong 2023	1	1	1	1	2	1	1
Kolovos 2023	1	1	1	1	1	1	1

© 2025 Tirta M et al. *JAMA Network Open*.

eTable 5. QUADAS-2 for Included Studies

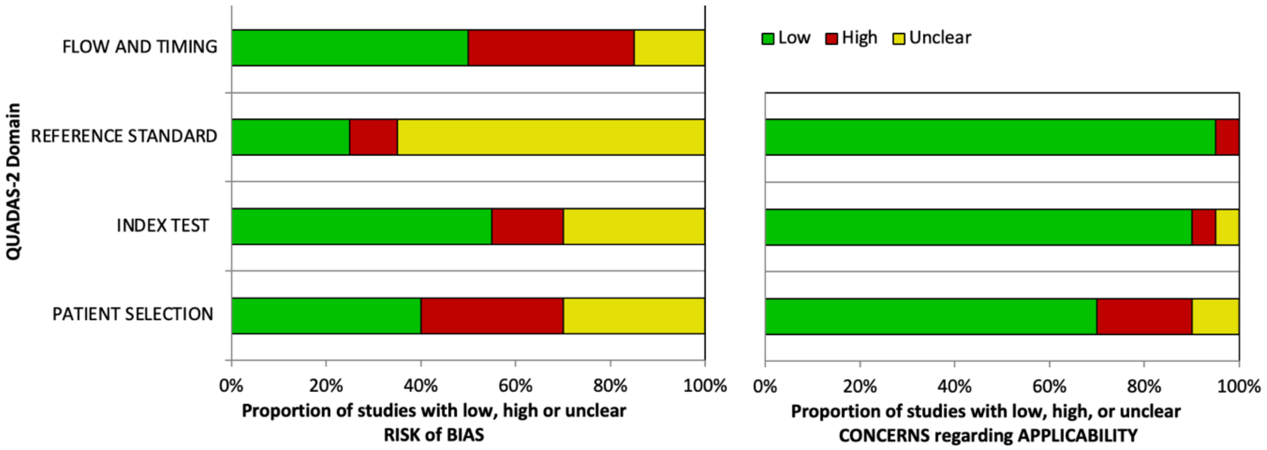

1 = low risk, 2 = high risk, 3 = unclear risk

Supplementary material Meta-analysis results

Primary analysis

eFigure 1. Forest Plot for Multiple Birth

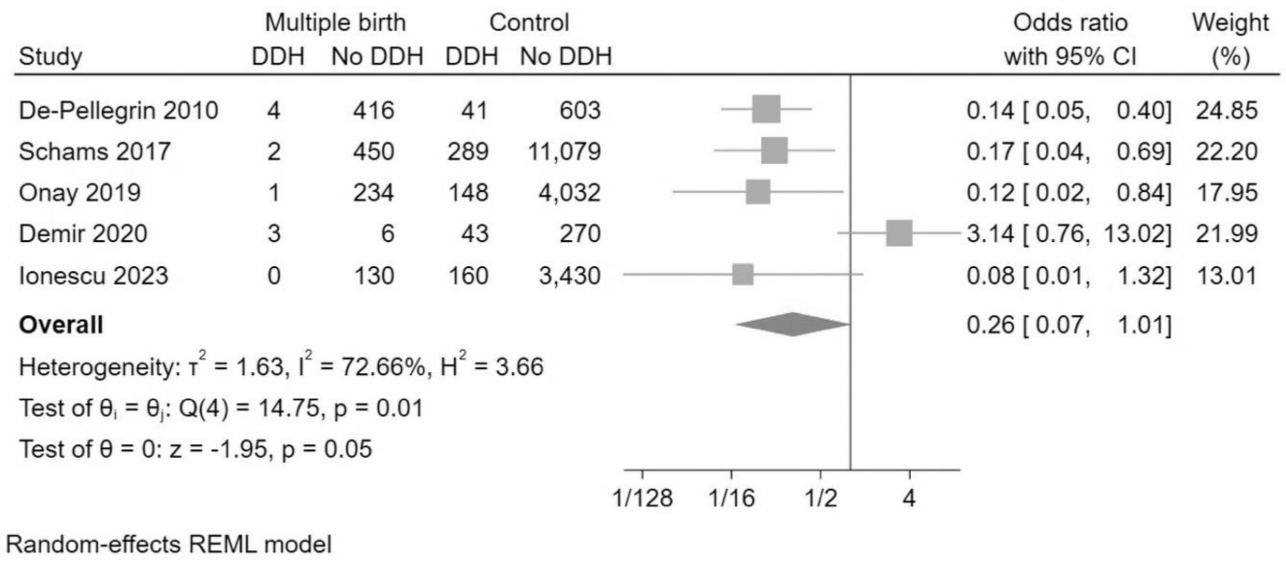

© 2025 Tirta M et al. *JAMA Network Open*.

eFigure 2. Forest Plot for Low Birth Weight

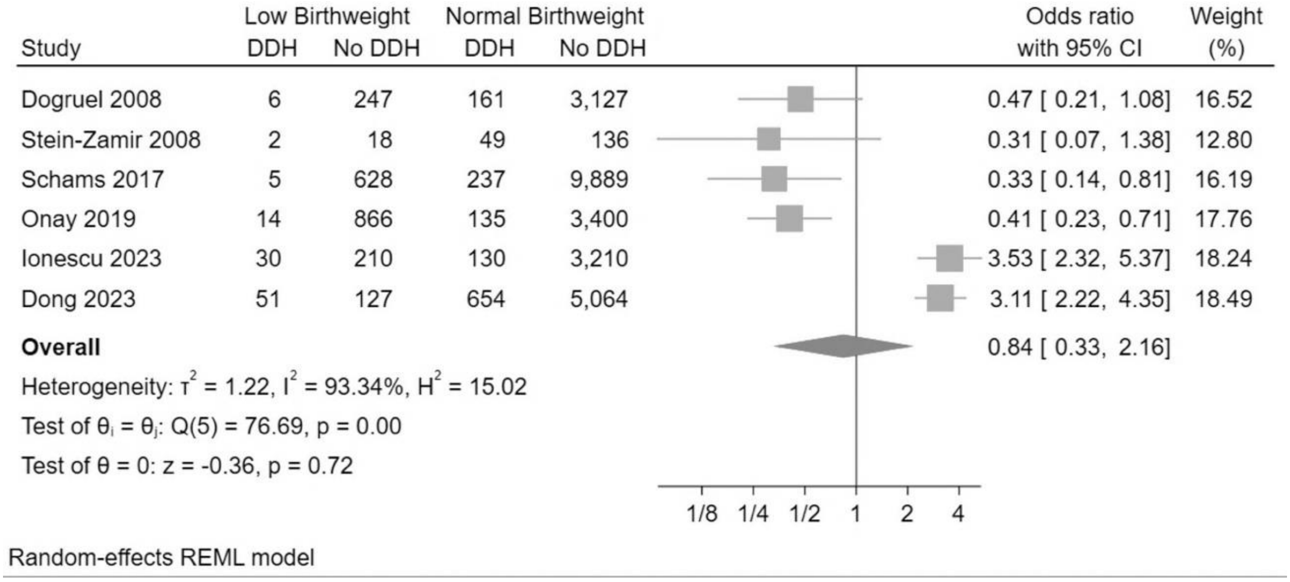

eFigure 3. Forest Plot for Cesarean Delivery

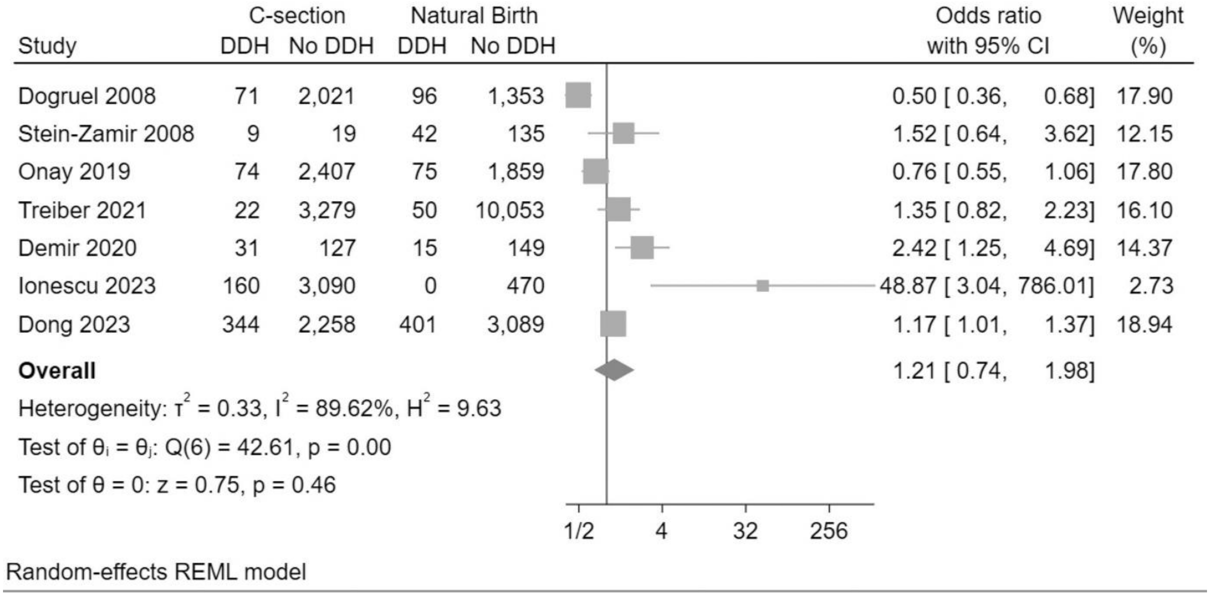

© 2025 Tirta M et al. *JAMA Network Open*.

eFigure 4. Forest Plot for Prematurity

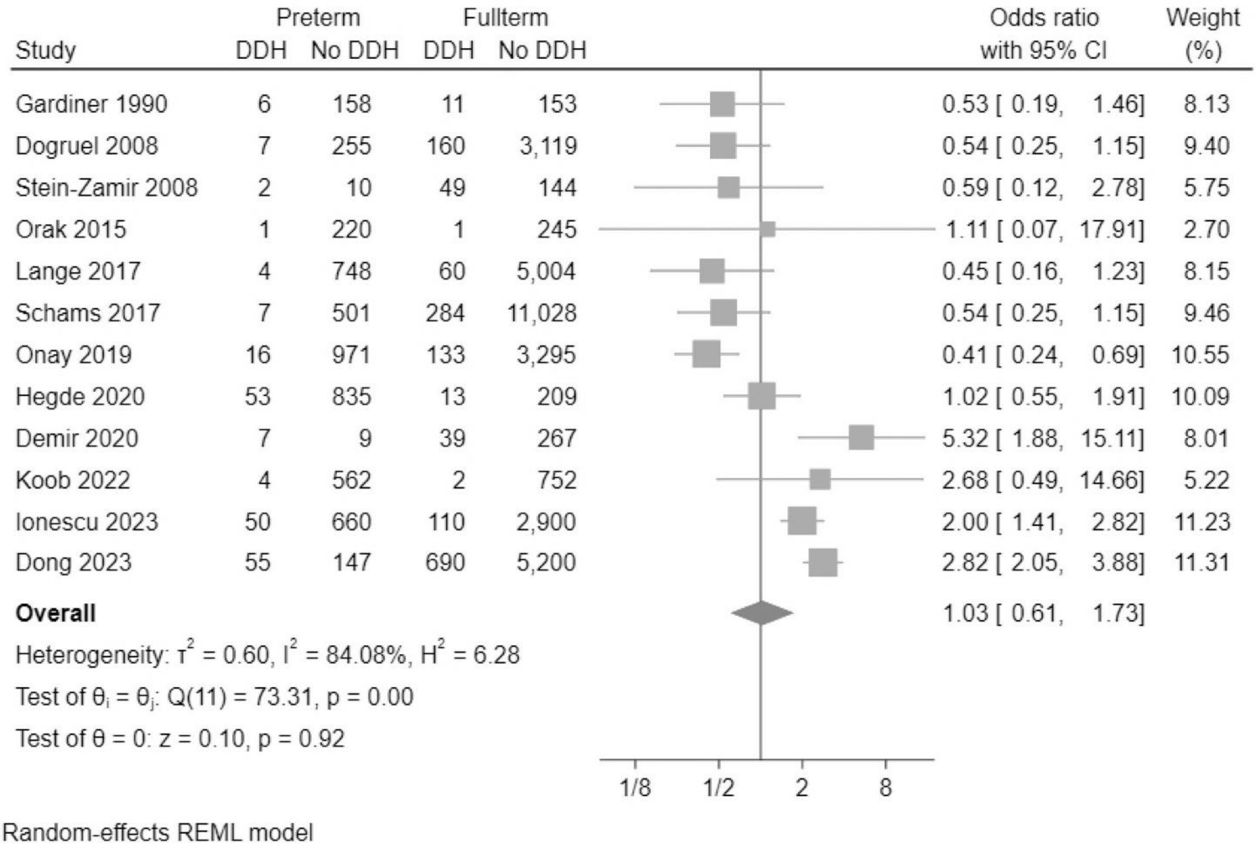

eFigure 5. Forest Plot for Primiparity (Firstborn Child)

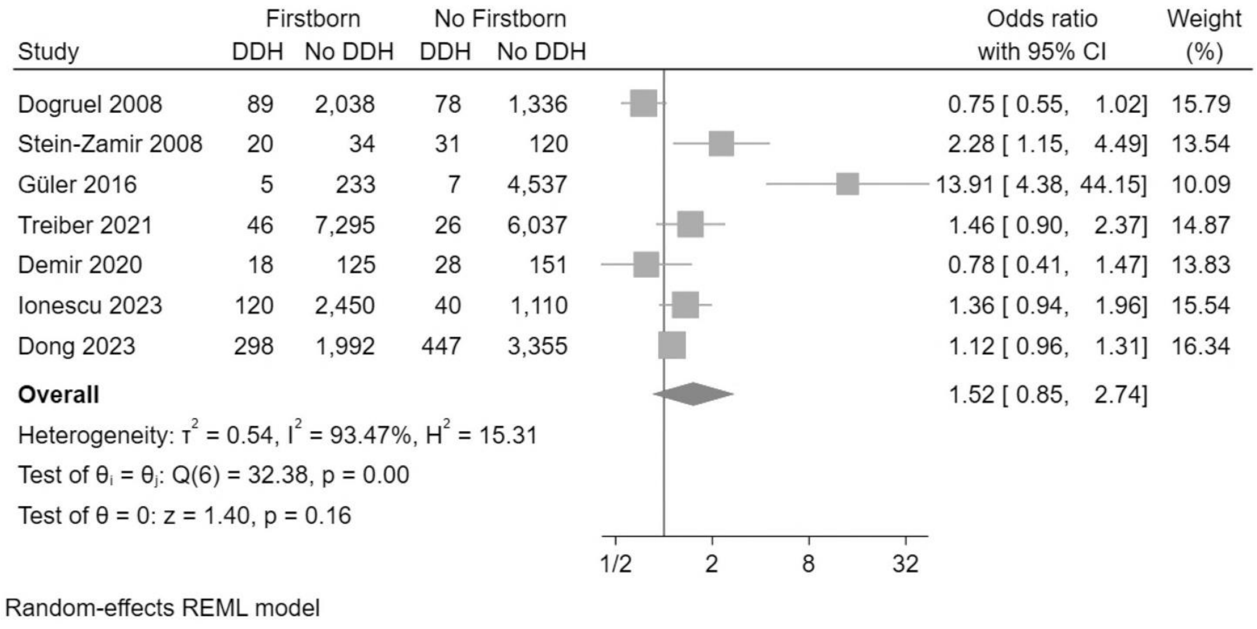

© 2025 Tirta M et al. *JAMA Network Open*.

eTable 6. Sensitivity Analysis for Different Risk Factors

Risk factor	n studies	OR	95% CI	Heterogeneity I^2
**Breech Presentation**				
Primary Analysis	15	4.15	2.62, 6.57	91.5%
Sensitivity analysis 1[1]	14	3.87	2.48, 6.03	91.3%
Sensitivity analysis 2[2]	12	4.63	2.98, 7.17	85.4%
Sensitivity analysis 3[3]	11	4.32	2.48, 7.54	94%
**Family history**				
Primary Analysis	10	3.83	2.05, 7.15	91.7%
Sensitivity analysis 1[2]	9	4.15	2.12, 8.14	92.5%
Sensitivity analysis 2[3]	8	3.93	1.88, 8.20	93.7%
Sensitivity analysis 3[4]	7	2.94	1.69, 5.11	87.1%
**Female sex**				
Primary Analysis	12	2.50	1.74, 3.59	88.2%
Sensitivity analysis 1[2]	10	2.75	1.87, 4.06	83.4%
Sensitivity analysis 2[3]	7	2.93	1.88, 4.55	92.3%
Sensitivity analysis 3[4]	5	3.87	2.70, 5.44	74.9%
**High birthweight**				
Primary Analysis	4	2.00	1.60, 2.49	0%
Sensitivity analysis 1[5]	3	1.96	1.55, 2.48	0%
**Oligohydramnios**				
Primary Analysis	5	3.76	1.66, 8.53	85.3%
Sensitivity analysis 1[2]	3	2.61	1.17, 5.83	48.4%
Sensitivity analysis 2[3]	4	4.01	1.45, 11.08	88.6%
**Multiple birth**				
Primary Analysis	5	0.26	0.07, 1.01	72.7%
Sensitivity analysis 1[2]	4	0.14	0.07; 0.29	0%
Sensitivity analysis 2[3]	3	0.14	0.05; 0.39	0%

[1] Without study Kolovos (Zero DDH cases in the control group)
[2] Only studies with counting per patients
[3] Only cohort studies
[4] Only universal screening
[5] Control group named as normal birthweight

Explanation: OR– Odds Ratio; 95% CI–95% confidence interval; I^2–inconsistency; τ^2–between-study variance.

Publication bias

eTable 7. Egger Regression Asymmetry Test for Risk Factor With 10 or More Studies Included in the Meta-Analysis

Risk Factors	Egger's Test
Breech presentation	p = 0.252
Family History	p = 0.479
Female sex	p = 0.580
Prematurity	p = 0.858

eFigure 6. Funnel Plot for Breech Presentation

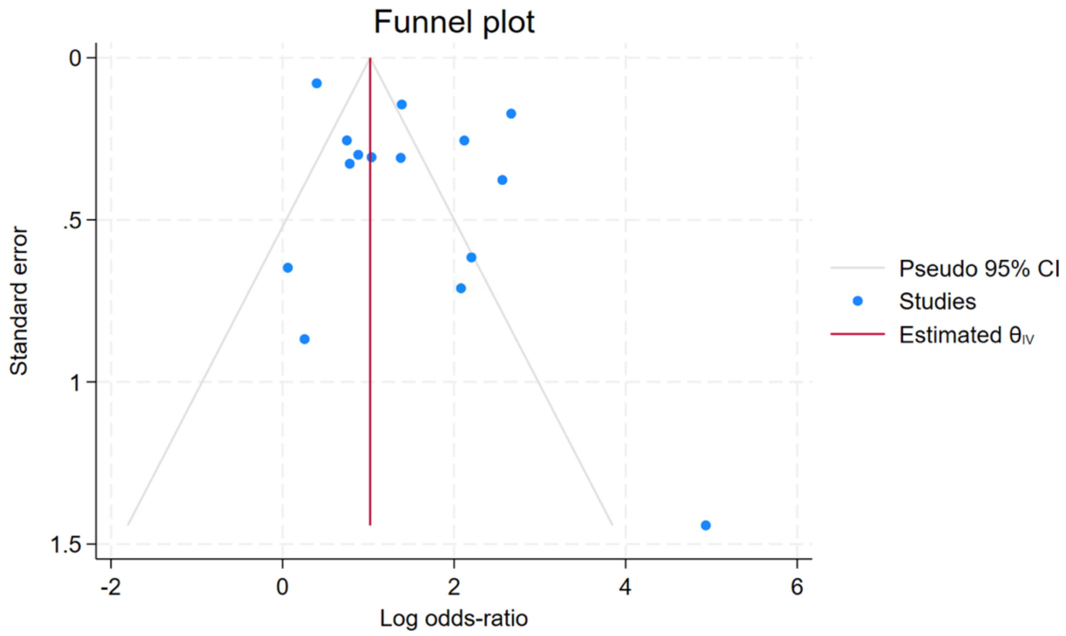

© 2025 Tirta M et al. *JAMA Network Open*.

eFigure 7. Funnel Plot for Family History

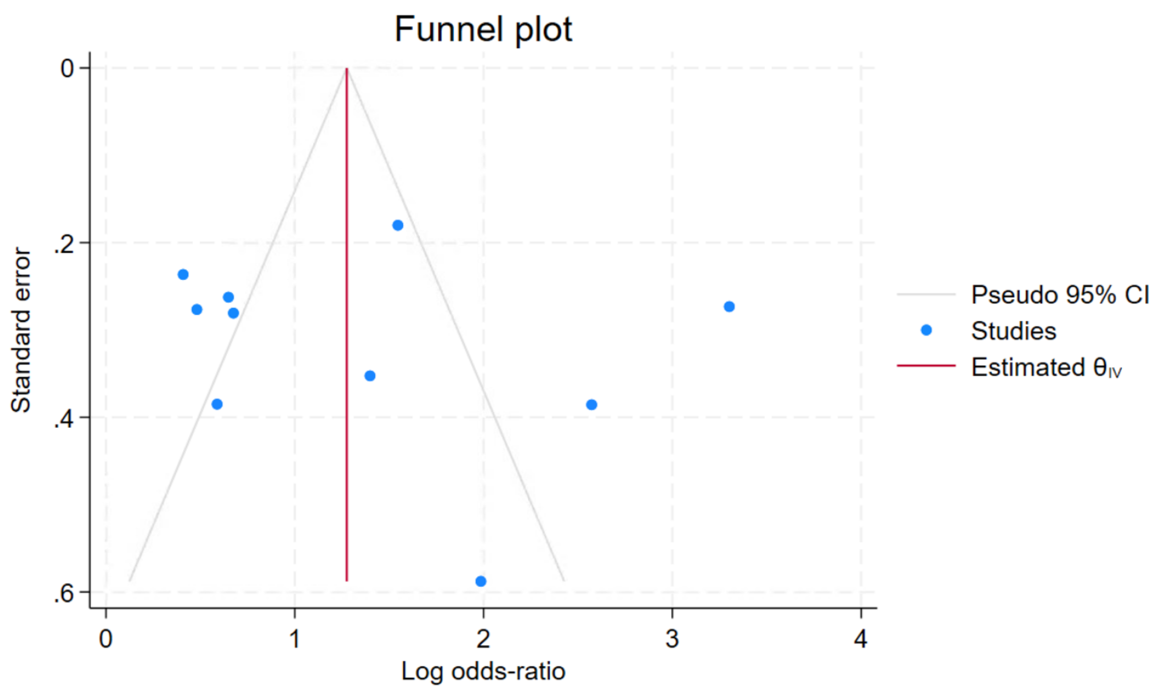

eFigure 8. Funnel Plot for Female Sex

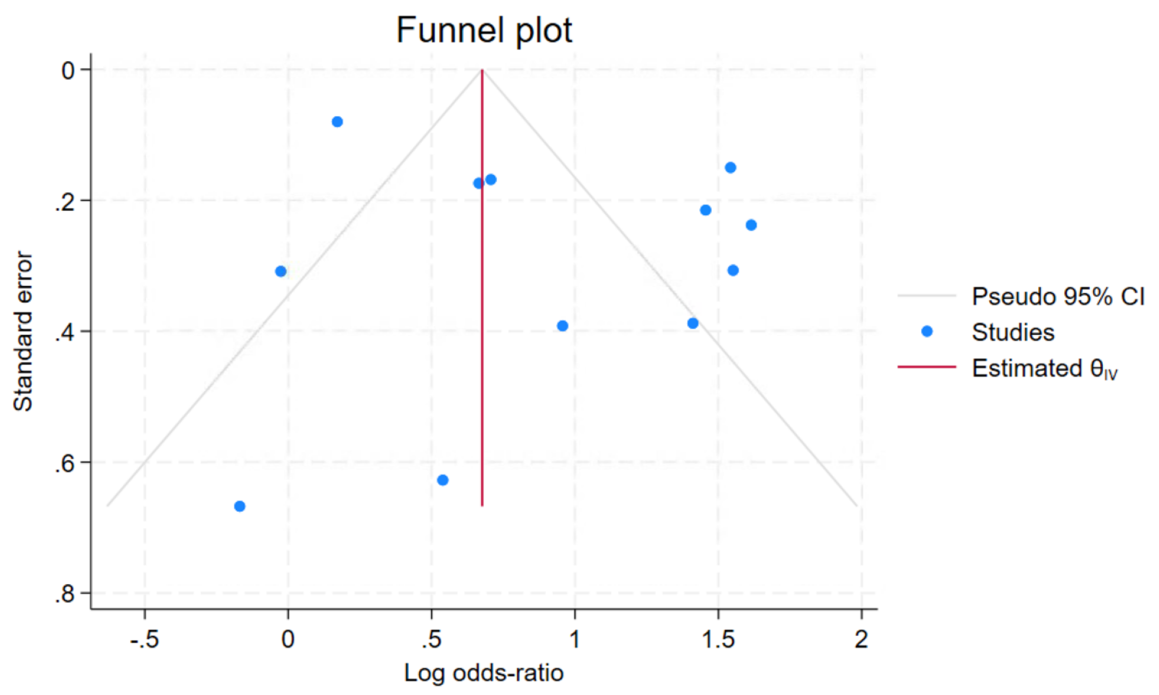

© 2025 Tirta M et al. *JAMA Network Open*.

eFigure 9. Funnel Plot for Prematurity

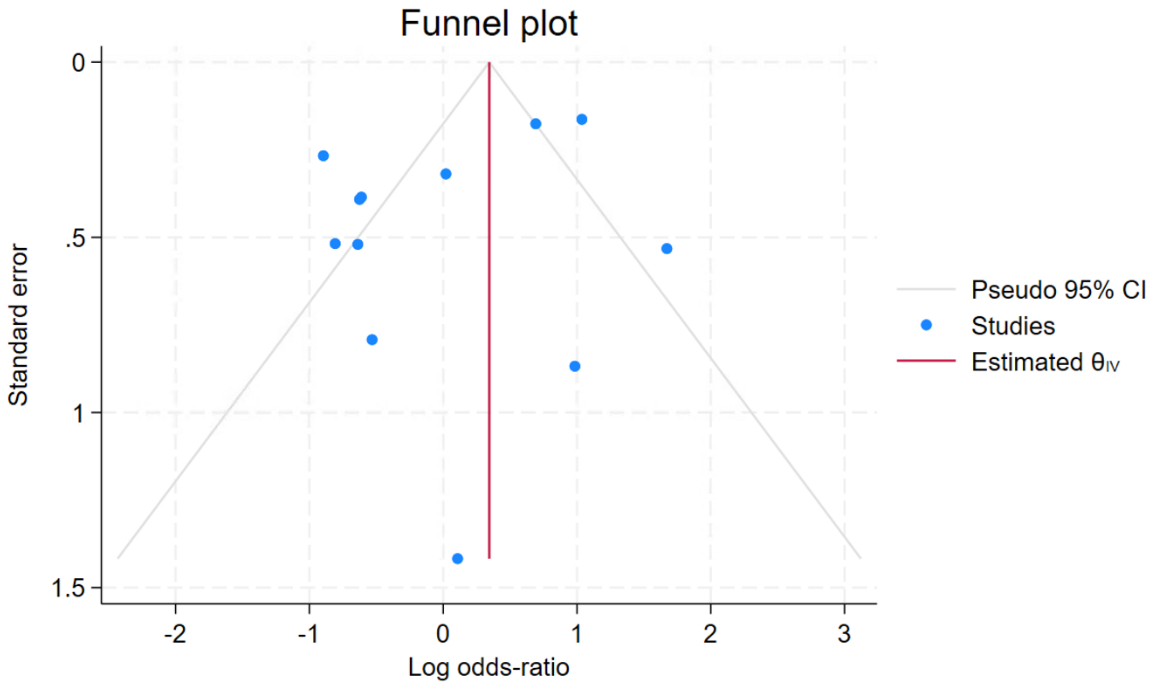

© 2025 Tirta M et al. *JAMA Network Open*.

eTable 8. Sensitivity and Subgroup Analysis

	Hips vs patients					
	Hips			Patients		
Risk factor	n studies	OR	95% CI	n studies	OR	95% CI
Breech presentation	3	4.51	0.29; 69.88	12	4.66	2.98; 7.17
C-section	2	1.57	0.78; 3.14	5	1.10	0.55; 2.18
Family history	1	1.80	0.85; 3.83	9	4.15	2.12; 8.14
Females	2	1.60	0.76; 3.38	10	2.75	1.87; 4.06
Firstborn	2	1.07	0.84; 1.37	5	1.94	0.83; 4.58
High birthweight	1	1.99	1.39; 2.84	3	2.01	1.62; 2.65
Low birthweight	1	3.11	2.22; 4.35	5	0.63	0.24; 1.66
Multiple births	1	3.14	0.76; 13.02	4	0.14	0.07; 0.29
Oligohydramnios	2	5.80	1.38; 24.43	3	2.61	1.17; 5.83
Prematurity	5	1.56	0.58; 4.17	7	0.78	0.45; 1.33

	Study design								
	Case-control			Cohort			Cross-sectional		
Risk factor	n studies	OR	95% CI	n studies	OR	95% CI	n studies	OR	95% CI
Breech presentation	3	2.79	1.10; 7.07	11	4.32	2.48; 7.54	1	9.04	2.7; 30.22
C-section	2	2.04	1.21; 3.45	5	0.99	0.59; 1.67	0	n/a	n/a
Family history	1	1.80	0.85; 3.83	8	3.93	1.88; 8.20	1	7.28	1.88; 8.20
Females	4	2.03	1.03; 4.00	7	2.93	1.88; 4.55	1	0.84	0.23; 3.12
Firstborn	2	1.32	0.46; 3.79	4	1.11	0.84; 1.46	1	13.91	4.38; 44.15
High birthweight	N/a	N/a	N/a	N/a	N/a	N/a	N/a	N/a	N/a
Low birthweight	1	0.31	0.07; 1.38	5	0.97	0.35; 2.74	0	N/a	N/a
Multiple births	2	0.64	0.03; 13.34	3	0.14	0.05; 0.39	0	N/a	N/a
Oligohydramnios	1	2.57	1.01; 6.56	4	4.01	1.45; 11.08	0	N/a	N/a
Prematurity	4	1.46	0.44; 4.80	8	0.90	0.51; 1.62	0	N/a	N/a

© 2025 Tirta M et al. *JAMA Network Open*.

	Risk of Bias								
	High			Low			Unclear		
**Risk factor**	**n studies**	**OR**	**95% CI**	**n studies**	**OR**	**95% CI**	**n studies**	**OR**	**95% CI**
Breech presentation	6	4.39	1.95; 9.86	4	3.70	1.14; 12.06	5	4.79	2.35; 9.79
C-section	4	2.03	0.49; 8.46	1	1.17	1.01; 1.37	2	0.98	0.56; 1.71
Family history	4	4.78	1.22; 18.76	2	3.06	1.27; 7.38	4	3.51	1.41; 8.72
Females	7	1.90	1.37; 2.63	3	2.98	1.17; 7.60	2	4.42	3.13; 6.25
Firstborn	5	1.74	0.68; 4.49	1	1.12	0.96; 1.31	1	1.46	0.90; 2.37
High birthweight	1	0.09	0.01; 1.41	2	2.00	1.58; 2.54	1	2.29	1.23; 4.26
Low birthweight	3	0.89	0.19; 4.09	2	1.06	0.12; 9.45	1	0.41	0.23; 0.71
Multiple births	3	0.37	0.04; 3.59	1	0.17	0.04; 0.69	1	0.12	0.02; 0.84
Oligohydramnios	3	2.03	1.19; 3.46	1	11.25	2.14; 13.33	1	4.60	2.14; 9.86
Prematurity	8	1.23	0.67; 2.29	2	1.28	0.26; 6.44	2	0.42	0.26; 0.66

	Time of screening								
	1 week			4-6 weeks			2-3 months		
**Risk factor**	**n studies**	**OR**	**95% CI**	**n studies**	**OR**	**95% CI**	**n studies**	**OR**	**95% CI**
Breech presentation	6	4.65	2.41; 8.96	4	2.77	1.67; 4.60	5	4.45	1.60; 12.41
C-section	1	1.35	0.82; 2.23	1	0.50	0.36; 0.68	5	1.47	0.83; 2.62
Family history	5	3.69	1.82; 7.45	2	3.02	0.65; 14.02	3	4.63	0.80; 26.71
Females	4	4.60	3.67; 6.77	3	1.36	0.75; 2.48	5	2.41	1.44; 4.03
Firstborn	1	1.46	0.90; 2.37	2	3.05	0.17; 53.43	4	1.22	0.96; 1.56
High birthweight	2	2.07	1.56; 2.74	0	N/a	N/a	2	0.57	0.03; 11.45
Low birthweight	1	0.33	0.14; 0.81	1	0.47	0.21; 1.08	4	1.19	0.34; 4.17
Multiple births	1	0.17	0.04; 0.69	1	0.14	0.05; 0.40	3	0.37	0.03; 4.13
Oligohydramnios	0	N/a	N/a	1	0.69	0.04; 11.6	4	4.24	1.84; 9.73
Prematurity	4	0.59	0.36; 0.97	2	0.77	0.41; 1.44	6	1.50	0.64; 3.53

© 2025 Tirta M et al. *JAMA Network Open*.

	Definition					
	>IIb			>IIc		
**Risk factor**	**n studies**	**OR**	**95% CI**	**n studies**	**OR**	**95% CI**
Breech presentation	11	4.26	2.33; 7.80	4	3.99	1.89; 8.43
C-section	N/a	n/a	n/a	N/a	n/a	n/a
Family history	6	4.84	1.79; 13.05	4	2.79	1.63; 4.77
Females	9	2.16	1.45; 3.23	3	4.59	3.59; 5.85
Firstborn	N/a	n/a	n/a	N/a	n/a	n/a
High birthweight	3	1.98	1.45; 2.69	1	2.02	1.48; 2.77
Low birthweight	5	1	0.35; 2.86	1	0.33	0.14; 0.81
Multiple births	4	0.29	0.05; 1.64	1	0.17	0.04; 0.69
Oligohydramnios	N/a	N/a	N/a	N/a	N/a	N/a
Prematurity	7	1.28	0.59; 2.78	5	0.72	0.47; 1.11

	Screening								
	Selective			Universal			N/a		
**Risk factor**	n studies	OR	95% CI	n studies	OR	95% CI	n studies	OR	95% CI
Breech presentation	4	5.94	1.95; 18.11	8	4.48	2.63; 7.62	3	1.98	0.97; 4.04
C-section	3	2.81	0.45; 16.83	3	0.78	0.45; 1.35	1	2.42	1.25; 4.69
Family history	2	15.59	4.36; 55.71	7	2.94	1.69; 5.11	1	1.80	0.85; 3.83
Females	4	1.74	0.98; 3.08	5	3.87	2.70; 5.44	3	1.56	0.79; 3.06
Firstborn	4	2.36	0.86; 6.43	2	1.02	0.53; 1.96	1	0.78	0.41; 1.47
High birthweight	2	0.57	0.03; 11.45	2	2.07	1.56; 2.74	0	N/a	N/a
Low birthweight	3	1.79	0.46; 6.91	3	0.40	0.27; 0.61	0	N/a	N/a
Multiple births	1	0.08	0.01; 1.32	2	0.15	0.05; 0.47	2	0.64	0.03; 13.34
Oligohydramnios	2	4.78	0.84; 27.13	2	2.96	0.62; 14.2	1	2.57	1.01; 6.56
Prematurity	5	2.22	1.57; 3.14	4	0.46	0.33; 0.66	3	1.39	0.38; 5.07

© 2025 Tirta M et al. *JAMA Network Open*.

Risk factor	Anticipated absolute effects (95% CI)		Relative effect (95% CI)	№ of participants (studies)	Certainty	What happens
	Risk with no risk factors	Risk with risk factors				
**Breech presentation**	25 per 1,000	**95 per 1,000** (62 to 143)	**OR 4.15** (2.62 to 6.57)	62750 (15 non-randomised studies)	⨁⨁⨁◯ Moderate [a,b]	Breech presentation increase the risk of DDH presence
**C-section**	38 per 1,000	**46 per 1,000** (29 to 73)	**OR 1.21** (0.74 to 1.98)	31699 (7 non-randomised studies)	⨁◯◯◯ Very low [a,b]	The evidence is very uncertain a bout the C-section effect on DDH
**Family History**	20 per 1,000	**73 per 1,000** (40 to 128)	**OR 3.83** (2.05 to 7.15)	53238 (10 non-randomised studies)	⨁⨁⨁◯ Moderate	Family history of DDH increase the risk of DDH presence
**Female sex**	21 per 1,000	**51 per 1,000** (36 to 72)	**OR 2.50** (1.74 to 3.59)	53138 (12 non-randomised studies)	⨁⨁◯◯ Low [a]	Female sex increase DDH presence, but with a moderate effect
**Firstborn**	38 per 1,000	**60 per 1,000** (32 to 98)	**OR 1.62** (0.85 to 2.74)	32066 (7 non-randomised studies)	⨁◯◯◯ Very low [a]	The evidence is very uncertain a bout the effect of the parity effect on DDH
**High birthweight**	34 per 1,000	**66 per 1,000** (54 to 81)	**OR 2.00** (1.60 to 2.49)	33985 (4 non-randomised studies)	⨁⨁◯◯ Low [b]	High birthweight may increase the risk of DDH presence
**Low Birthweight**	52 per 1,000	**44 per 1,000** (18 to 106)	**OR 0.84** (0.33 to 2.16)	28396 (6 non-randomised studies)	⨁◯◯◯ Very low [a]	The evidence is very uncertain a bout the low birthweight effect on DDH
**Multiple births**	34 per 1,000	**9 per 1,000** (2 to 34)	**OR 0.26** (0.07 to 1.01)	21341 (5 non-randomised studies)	⨁⨁⨁◯ Moderate	The evidence suggests that multi ple birth results in little to no effect on DDH
**Oligohydramnios**	45 per 1,000	**151 per 1,000** (73 to 288)	**OR 3.76** (1.66 to 8.53)	18090 (5 non-randomised studies)	⨁⨁◯◯ Low [a]	The evidence suggests that oligo hydramnios results in an increase in DDH presence
**Prematurity**	46 per 1,000	**47 per 1,000** (28 to 77)	**OR 1.03** (0.61 to 1.73)	39156 (12 non-randomised studies)	⨁⨁◯◯ Low	The evidence suggests that prem aturity does not increase DDH presence
GRADE Working Group grades of evidence<br>High certainty: We are very confident that the true effect lies close to that of the estimate of the effect.<br>Moderate certainty: We are moderately confident in the effect estimate: the true effect is likely to be close to the estimate of the effect, but there is a possibility that it is substantially different.<br>Low certainty: Our confidence in the effect estimate is limited: the true effect may be substantially different from the estimate of the effect.<br>Very low certainty: We have very little confidence in the effect estimate: the true effect is likely to be substantially different from the estimate of effect.						

eTable 9. Summary of Findings With GRADE System of Evidence

a. High risk of bias for half or more studies
b. One study extreme OR

The number of "⨁" shows the grade of evidence—one cross is very low, two is low and so on.

© 2025 Tirta M et al. *JAMA Network Open*.
