# Supplementary material for: Risk Factors for Developmental Dysplasia of the Hip Before 3 Months of Age: A Meta-Analysis
Source: JAMA Netw Open. 2025 Jan 24;8(1):e2456153. doi: 10.1001/jamanetworkopen.2024.56153 (PMC11762239; doi:10.1001/jamanetworkopen.2024.56153)
Supplement: Supplement 2. — Data Sharing Statement [file jamanetwopen-e2456153-s002.pdf]

Data Sharing Statement

Tirta. Risk Factors for Developmental Dysplasia of the Hip Before 3 Months of Age. *JAMA Netw Open*. Published January 24, 2025. doi:10.1001/jamanetworkopen.2024.56153

Data
Data available: Yes
Data types: Data (not involving human participants)
How to access data: Supplementary Material
When available: With publication

Supporting Documents
Document types: Other (please specify)
Additional Information: Supplementary Material
How to access documents: Supplementary Material
When available: With publication

Additional Information
Who can access the data: Supplementary Material
Types of analyses: For any purpose
Mechanisms of data availability: Supplementary Material
